# Photocatalytic Atom Transfer Radical Addition to Olefins Utilizing Novel Photocatalysts

**DOI:** 10.3390/molecules24091644

**Published:** 2019-04-26

**Authors:** Errika Voutyritsa, Ierasia Triandafillidi, Nikolaos V. Tzouras, Nikolaos F. Nikitas, Eleftherios K. Pefkianakis, Georgios C. Vougioukalakis, Christoforos G. Kokotos

**Affiliations:** Laboratory of Organic Chemistry, Department of Chemistry, National and Kapodistrian University of Athens, Panepistimiopolis 15771, Athens, Greece; errikav@chem.uoa.gr (E.V.); iertriant@chem.uoa.gr (I.T.); nitzouras@gmail.com (N.V.T.); niknikitas@chem.uoa.gr (N.F.N.); pefkos99ele@gmail.com (E.K.P.)

**Keywords:** photocatalysis, ATRA reactions, olefins, sustainable catalysis, ruthenium-based complexes

## Abstract

Photocatalysis is a rapidly evolving area of research in modern organic synthesis. Among the traditional photocatalysts, metal-complexes based on ruthenium or iridium are the most common. Herein, we present the synthesis of two photoactive, ruthenium-based complexes bearing pyridine-quinoline or terpyridine ligands with extended aromatic conjugation. Our complexes were utilized in the atom transfer radical addition (ATRA) of haloalkanes to olefins, using bromoacetonitrile or bromotrichloromethane as the source of the alkyl group. The tailor-made ruthenium-based catalyst bearing the pyridine-quinoline bidentate ligand proved to be the best-performing photocatalyst, among a range of metal complexes and organocatalysts, efficiently catalyzing both reactions. These photocatalytic atom transfer protocols can be expanded into a broad scope of olefins. In both protocols, the photocatalytic reactions led to products in good to excellent isolated yields.

## 1. Introduction

Photocatalysis, the use of light to promote organic transformations, is a rather old concept that was first conceived in the early 1900s by Ciamician [1]. Since then, Photocatalysis spent almost a century receiving limited attention from researchers, as scientists were preoccupied with the thought that radical chemistry is rather difficult to be controlled. In 2008, Macmillan [2], Yoon [3] and Stephenson [4] put forward the idea of photoredox catalysis, where transition-metal complexes could be employed as photocatalysts, under mild reaction conditions. Since then, the field of Photocatalysis has expanded significantly [5,6,7,8,9,10,11,12]. 

Olefins are very common building blocks in synthetic organic chemistry, due to their availability and wide participation in addition reactions, leading to highly-useful products in a controlled fashion. Also, alkyl halides are commonly employed in organic synthesis, since they bear a reactive carbon-halogen bond, which can be homolytically cleaved with the use of radical initiators or photocatalysts. Atom transfer radical addition (ATRA) of haloalkanes to unsaturated compounds serves as a simple and useful method to form C-C and C-X bonds. Many successful ATRA reactions have been reported in literature, most of which are metal-catalyzed, employing copper-, ruthenium-, iridium-, palladium-, or nickel-based complexes [13,14,15,16,17,18]. Along the same lines, Bach, Nicewicz and Melchiorre emphasized the ease of controlling photogenerated radicals, opening new avenues in the field of photocatalysis [19,20,21].

**Scheme 1:**
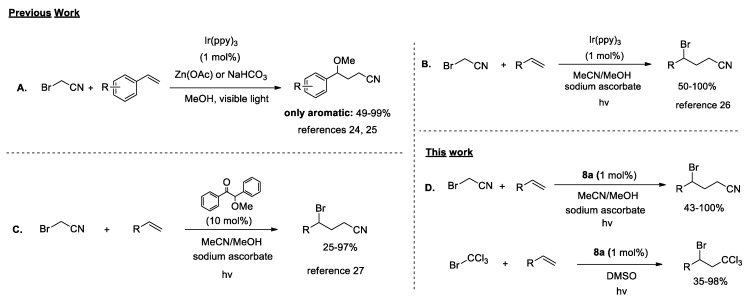
Photocatalytic methodologies of atom transfer radical addition (ATRA) reactions with bromoacetonitrile, (**A**) previous work from other groups, (**B**) previous work from our group with an iridium photocatalyst, (**C**) previous work from our group with a photoinitiator, (**D**) this work.

Brominated compounds are valuable scaffolds in organic synthesis, being able to undergo further transformation into useful functional groups. A well-known brominating reagent for ATRA reactions is bromoacetonitrile, which has been widely used, by different research groups, making it a well-established precursor of addition products [22,23,24,25]. However, the scope of olefins employed in ATRA reactions with bromoacetonitrile, in the presence of a metal-based catalyst, is rather limited (Scheme 1A). We have already contributed to this field with two studies, one focusing on expanding the scope of olefins with iridium photocatalysts, just by altering the reaction mechanism utilizing sodium ascorbate [26] (Scheme 1B) and by introducing a photoorganocatalytic alternative [27] (Scheme 1C). The synthesis and study of new metal-based complexes that can be employed as photocatalysts is essential, in order to further expand the substrate scope. Moreover, in order to widen the scope of brominated addition products, the use of other alkyl halides, such as bromotrichloromethane, which is used successfully in ATRA reactions, is required [28,29,30].

Using our experience in Photocatalysis [26,27,31,32,33,34] and combining it with the synthesis of ruthenium-based photoactive complexes [35,36,37,38,39], we present the use of alternative ruthenium-based photocatalysts, rather than the well-known and commercially available [Ru(bpy)_3_]Cl_2_, in the ATRA reaction of bromoacetonitrile and bromotrichloromethane with olefins bearing various functional groups (Scheme 1D). 

These ruthenium photocatalysts bear bidentate pyridine-quinoline hybrid ligands or tridentate terpyridine ligands with extended aromatic conjugation and anthracene units attached to them. Such architectures can increase the extinction coefficients of the metal-to-ligand charge transfer bands in the corresponding complexes, as well as their response to red light, as previously found in dye-sensitized solar and singlet oxygen sensitization applications [36,37,38,39]. Moreover, as shown, fundamental photophysical studies of these and analogous scaffolds, the anthracene moiety acts as a secondary chromophore/quencher, thus improving the sensitizing ability of the corresponding complexes [37,39].

## 2. Results

### 2.1. Synthesis of Photocatalysts

**Scheme 2:**
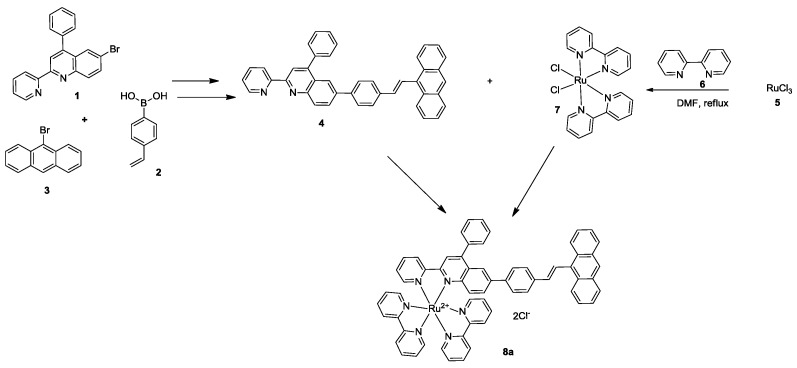
Synthesis of complex 8a.

In this work, we report the synthesis of two ruthenium complexes and study their activity as photocatalysts in ATRA reactions. The synthesis of the first ruthenium complex started with the Suzuki reaction between pyridine-quinoline 1, boronic acid 2 and bromoanthracene 3 affording ligand 4. In parallel, RuCl_3_ reacted with bipyridine 6 in DMF (dimethyl formamide) to give complex 7 in 62% yield. Finally, complex 7 reacted with bipyridine 5 in ethylene glycol, leading to the desired 8a (Scheme 2) [36,37,39].

**Scheme 3:**
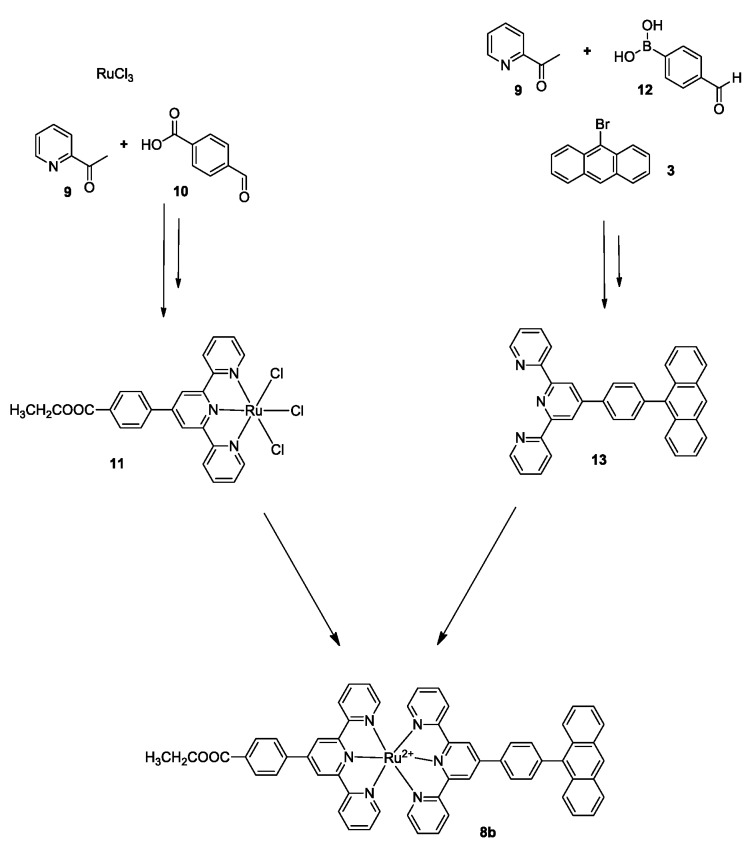
Synthesis of complex 8b.

For the synthesis of 8b, acetyl pyridine 9 reacted with acid 10 and RuCl_3_ furnishing compound 11 (Scheme 3). In parallel, acetyl pyridine 9 reacted with boronic acid 12 and the bromoanthracene 3 afforded compound 13. Finally, complex 13 reacted with complex 11, in the presence of *N*-methylmorpholine, leading to the final complex 8b in 85% yield (Scheme 3) [35,38].

### 2.2. Photocatalytic Reactions

As mentioned earlier, the ATRA reaction between olefins and bromoacetonitrile is a very useful organic transformation that can be used as a benchmark reaction for testing new photocatalysts. The ruthenium and iridium complexes previously described in literature were employed in this ATRA reaction, providing very good results. We began our studies with the reaction between 1-decene and bromoacetonitrile (Table 1). Commercially available photocatalysts, Ir(ppy)_3_ (8c) [26] and Ru(bpy)_3_Cl_2_ (8d) [2,3,4] were tested, for comparison purposes (Table 1, entries 1 and 2). By utilizing only 1 mol% catalyst loading, iridium complex 8c, the most expensive of the two commercially available metal-based photocatalysts tested, allowed a product formation in a quantitative yield, while ruthenium complex 8d led to a diminished 78% yield. A range of photocatalysts were then tested, with our tailor-made ruthenium-based photocatalyst having the pyridine-quinoline ligand of extended aromatic conjugation 8a affording very good yields (Table 1, entries 3, 4, 8, 9). It has to be noted that 8a outperformed the commercially available Ru(bpy)_3_Cl_2_ catalyst, being slightly inferior in catalytic efficiency compared to 8d (iridium-based complex) (Table 1, entry 3 vs. 2). Studying the reaction conditions, the use of sodium ascorbate proved to be essential (Table 1, entry 5). Also, when the reaction was kept in dark, no conversion was detected (Table 1, entry 6). Unlike previous literature precedent [24,25], no alcoholic addition took place. We believe that the use of sodium ascorbate alters the reaction mechanism, bypassing this reaction route. 

**Scheme 4:**
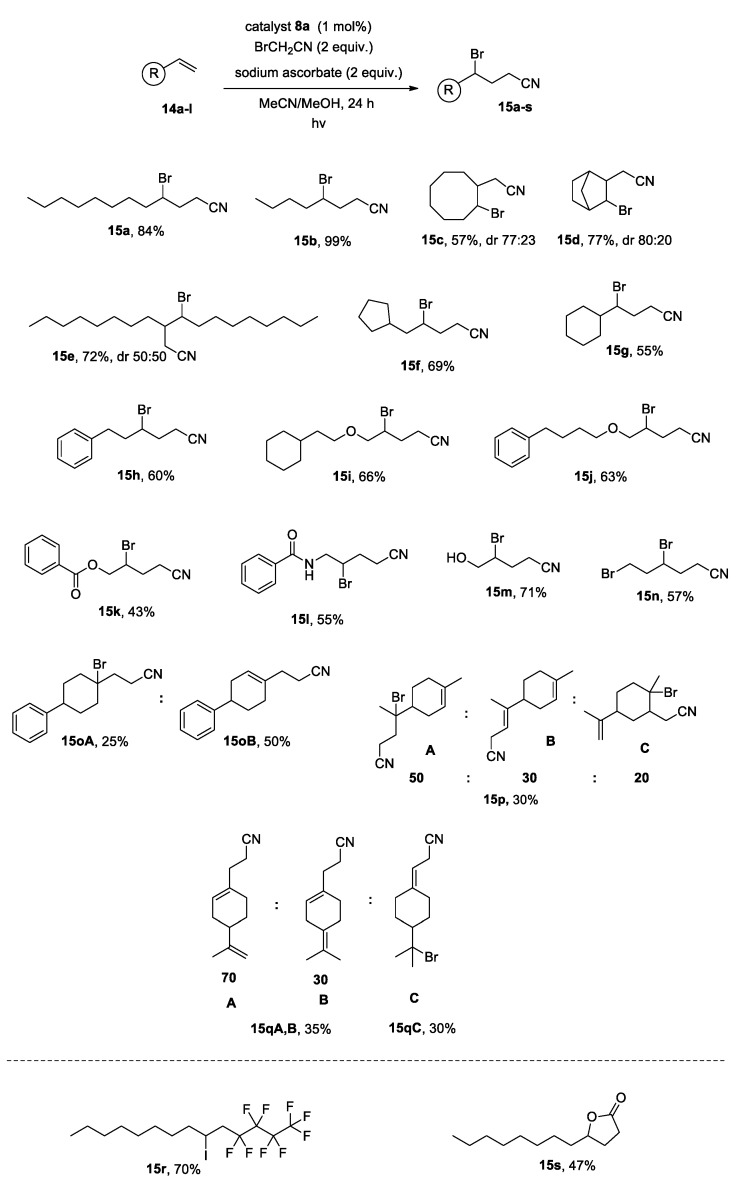
Substrate scope of the photocatalytic reaction between bromoacetonitrile and olefins.

Following the optimization of the reaction conditions, we tested the substrate scope of the reaction, as far as the aliphatic olefin is concerned (Scheme 4). All types of double bonds tested led to the corresponding addition products in good to excellent isolated yields. Initially, aliphatic double bonds were tested, leading to the desired products in very good to excellent yields (Scheme 4, 15a, 15b, 15e–h). The usually less reactive disubstituted double bonds, like cyclooctene (14c) and norbornene (14d), were also examined (Scheme 4, 15c and 15d), providing very good results. Furthermore, allyl ethers proved to be efficient substrates for this type of atom transfer radical addition as well, since the desired products were obtained in very good yields (Scheme 4, 15i and 15j). Finally, substrates bearing different functional groups, such as ester (Scheme 4, 15k), amide (Scheme 4, 15l), free alcohol (Scheme 4, 15m) and halogens (Scheme 4, 15n), were tested, affording the corresponding products in good to very good yields. Also, the 1,1-disubstituted double bond 4-methylenecyclohexyl)benzene (14o) was examined furnishing two products (Scheme 4, 15oA and 15oB), where the main product was the product of elimination of HBr. Trisubstituted naturally occurring double bonds, like limonene (14p) and β-pinene (14q) were also tested on this protocol, furnishing various products (Scheme 4, 15p and 15q). In the case of limonene, the result of the reaction was a mixture of three products. Regarding β-pinene, the reaction afforded two different fractions, where the first was a mixture of two compounds (15qA and 15qB) and compound 15qC. During the optimization process, the use of other addition reagents, such as iodoacetic acid and nonafluoro-1-iodobutane, was also examined. The reaction furnished the corresponding product of addition to 1-decene (Scheme 4, 15r and 15s), but in lower yields than the additional product of bromoacetonitrile (Scheme 4, 15a), so it was a rational choice to continue further with the latter.

**Scheme 5:**
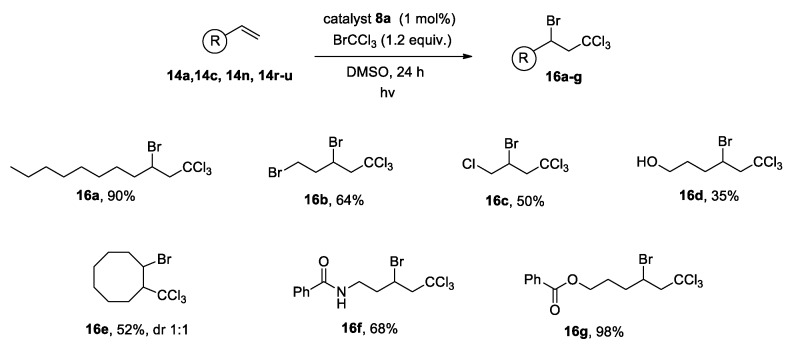
Substrate scope of the photocatalytic reaction between bromotrichloromethane and olefins.

In a similar manner, we also studied the reaction between bromotrichloromethane and aliphatic olefins (Scheme 5). The reaction conditions employed were adopted from the literature [40]. Using these conditions, we tested a number of substrates (Scheme 5). When 1-decene was employed, the targeted product was obtained in excellent yield (Scheme 5, 16a). A more challenging disubstituted alkene, cyclooctene, was tested and proved to be a competent substrate, affording the desired product in good yield (Scheme 5, 16e). Olefins bearing different functional groups were also studied and gave the corresponding products in very good yields (Scheme 5, 16b–d, 16f, 16g).

### 2.3. Mechanistic Studies

#### 2.3.1. Phosphorescence Quenching Studies

**Scheme 6:**
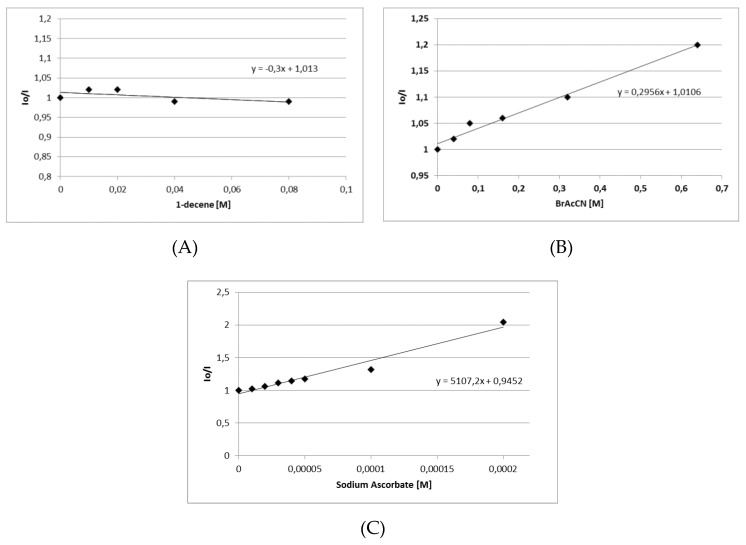
(**A**) Phosphorescence quenching studies of 8a with 1-decene. (**B**) Phosphorescence quenching studies of 8a with bromoacetonitrile. (**C**) Phosphorescence quenching studies of 8a with sodium ascorbate.

To shed light on the mechanism of these transformations, a series of phosphorescence quenching studies were carried out (Scheme 6). Regarding the reaction with bromoacetonitrile, after irradiation of the photocatalyst at 370 nm, its phosphorescence was measured at 516 nm. Upon increasing the amount of the added 1-decene, no changes in the phosphorescence were observed (Scheme 6A). Increasing the amount of the added bromoacetonitrile, a constant decrease in the phosphorescence was observed (Scheme 6B). Finally, increasing the amount of the added sodium ascorbate, a constant decrease in the phosphorescence was also observed (Scheme 6C). From these data, we conclude that sodium ascorbate is important for the success of the reaction and is a better quencher than bromoacetonitrile.

**Scheme 7:**
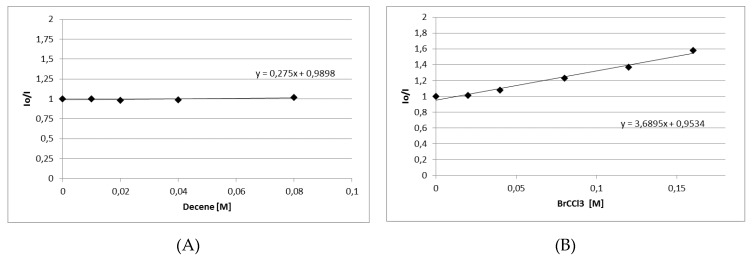
(**A**) Phosphorescence quenching studies of 8a with 1-decene. (**B**) Phosphorescence quenching studies of 8a with bromotrichloromethane.

Regarding the reaction with bromotrichloromethane, the same phosphorescence quenching experiments were carried out (Scheme 7). After irradiation of 8a at 370 nm, its phosphorescence was measured 516 nm. When the amount of the added 1-decene was increased, no change in phosphorescence was observed (Scheme 7A). Increasing the amount of the added bromotrichloromethane, a constant decrease in the phosphorescence was observed (Scheme 7B).

#### 2.3.2. Quantum Yield Measurement

**Scheme 8:**
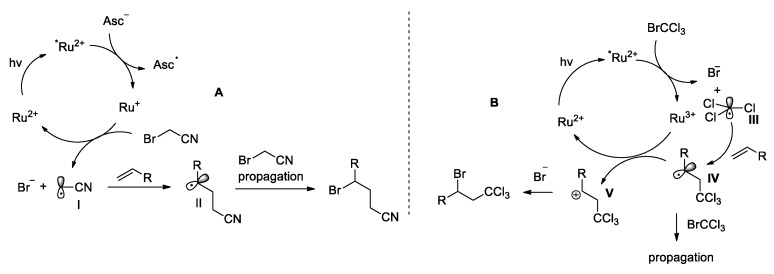
Proposed reaction mechanism.

In an effort to further understand the mechanism of the reaction, we also measured the quantum yield of both reactions, in order to discover if they progress through a closed photocatalytic cycle or via a propagation mechanism [41]. The quantum yield of both reactions proved to be higher than 1, (Φ_1_ = 9 and Φ_2_ = 3), indicating that in both reactions, a propagation mechanism was taking place. On the basis of these data, we propose the mechanisms presented in Scheme 8. Both of these proposed catalytic cycles begin with the excitation of the photocatalyst after the absorption of visible light irradiation. The following step is the one that differentiates the two cycles. In the presence of an electron donor, such as sodium ascorbate, the left photocatalytic cycle occurs (Scheme 8A). The cycle begins with the reductive quenching of the photocatalyst by sodium ascorbate, leading to Ru^+^, followed by a single electron transfer (SET) between the photocatalyst and bromoacetonitrile, leading to alkyl radical I. Radical I will subsequently react with the double bond, giving a second radical species II, which via propagation leads to product formation. In the second cycle (B), in the first step, the excited Ru^2+^ catalyst will participate in an oxidative quenching through a SET procedure, resulting to the formation of radical III. Then, radical III will react with the olefin, furnishing radical species IV. In this step, radical IV will either lead to the propagation route or can participate to a SET with the ruthenium photocatalyst leading to carbocation V, which will be attacked by a bromine anion giving the final product (Scheme 8B). However, quantum yield experiments suggest that the first cycle is more likely taking place.

Synthetic procedures for the preparation of the photocatalysts, reaction optimization, compound characterization data, ^1^H- and ^13^C-NMR of compounds, full mechanistic studies including UV-Vis and phosphorescence studies can be found at Supplementary Materials.

## 3. Conclusions 

In conclusion, we report the synthesis of two ruthenium complexes, the unprecedented evaluation of their photocatalytic efficiency in two well-known ATRA reactions, and the synthesis of a wide variety of brominated products in good to excellent yields. The reactions took place under mild conditions, using common household lamps and no inert atmosphere, with no need for dry solvent, and employing a low catalyst loading. Mechanistic experiments were carried out, shedding some light on the mechanism of the reactions.

## Figures and Tables

**Table 1:**
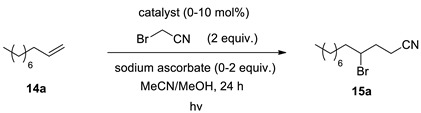
Optimization of the reaction conditions for the photocatalytic atom transfer radical addition of bromoacetonitrile to olefins.

Entry	Catalyst	Catalyst Loading (mol %)	Sodium Ascorbate (Equiv.)	Yield (%) ^a^
1	Ir(ppy)_3_ (**8c**)	1	2	98
2	Ru(bpy)_3_Cl_2_ (**8d**)	1	2	78
**3**	**8a**	**1**	**2**	**84**
4	**8b**	1	2	63
5	**8a**	1	-	0
6 ^b^	**8a**	1	2	0
7	-	-	2	0
8	Thioxanthone (**8e**)	10	2	43
9	Eosin Y (**8f**)	10	2	32

^a^ Isolated yield. The reaction was performed with 1-decene (35 mg, 0.25 mmol), bromoacetonitrile (60 mg, 0.50 mmol), catalyst (0–10 mol%, 0.00–0.025 mmol), sodium ascorbate (0–100 mg, 0–0.50 mmol), acetonitrile (2 mL) and methanol (1.5 mL) under household bulb irradiation for 24 h. ^b^ The reaction was performed in the dark.

## Supplementary Materials

The following are available online at https://www.mdpi.com/1420-3049/24/9/1644/s1, Synthetic procedures for the preparation of the photocatalysts, reaction optimization, compound characterization data, ^1^H- and ^13^C-NMR of compounds, full mechanistic studies including UV-Vis and phosphorescence studies.
