# Supplementary material for: Photocatalytic Atom Transfer Radical Addition to Olefins Utilizing Novel Photocatalysts
Source: Molecules. 2019 Apr 26;24(9):1644. doi: 10.3390/molecules24091644 (PMC6540230; doi:10.3390/molecules24091644)
Supplement: Supplementary file 1 [file molecules-24-01644-s001.pdf]

Photocatalytic Atom Transfer Radical Addition to Olefins utilizing Novel Photocatalysts

Errika Voutyritsa, Ierasia Triandafillidi, Nikolaos V. Tzouras, Nikolaos F. Nikitas, Eleftherios K. Pefkianakis, Georgios C. Vougioukalakis* and Christoforos G. Kokotos*

Laboratory of Organic Chemistry, Department of Chemistry, National and Kapodistrian University of Athens,

Panepistimiopolis, Athens 15771, Greece

SUPPORTING INFORMATION

	Page
**General Remarks**	**S2**
**Optimization of the Reaction Conditions for the Photocatalytic Reaction between 1-decene and $BrCH_2CN$**	**S3**
**Synthesis of Photocatalysts**	**S5**
**Synthesis of the Starting Materials**	**S12**
**General Procedure for the Photocatalytic Reaction between Olefins and $BrCH_2CN$**	**S18**
**General Procedure for the Photocatalytic Reaction between Olefins and $BrCCl_3$**	**S29**
**Determination of the Quantum Yield**	**S33**
**Phosphorescence Quenching Studies**	**S36**
**References**	**S41**
**NMR Spectra**	**S43**

General Remarks

Chromatographic purification of products was accomplished using forced-flow chromatography on Merck® Kieselgel 60 F_{254} 230-400 mesh. Thin-layer chromatography (TLC) was performed on aluminum backed silica plates (0.2 mm, 60 F_{254}). Visualization of the developed chromatogram was performed by fluorescence quenching, using phosphomolybdic acid, anisaldehyde or potassium permanganate stains. Mass spectra (ESI) were recorded on a Finningan® Surveyor MSQ LC-MS spectrometer. HRMS spectra were recorded on Bruker® Maxis Impact QTOF spectrometer. ^{1}H and ^{13}C NMR spectra were recorded on Varian® Mercury (200 MHz and 50 MHz respectively), or a Bruker® Avance (500 MHz and 125 MHz), and are internally referenced to residual solvent signals. Data for ^{1}H NMR are reported as follows: chemical shift (δ ppm), integration, multiplicity (s = singlet, d = doublet, t = triplet, q = quartet, m = multiplet, br s = broad singlet), coupling constant and assignment. Data for ^{13}C NMR are reported in terms of chemical shift (δ ppm). Mass spectra and conversions of the reactions were recorded on a Shimadzu® GCMS-QP2010 Plus Gas Chromatograph Mass Spectrometer utilizing a MEGA® column (MEGA-5, F.T.: 0.25 μm, I.D.: 0.25 mm, L: 30 m, T_{max}: 350 ^{o}C, Column ID# 11475). A Varian® Cary 50 UV-Vis spectrophotometer was used for the quantum yield measurements. A Scinco® FS-2 fluorescence spectrometer was used for the phosphorescence quenching studies.

Optimization of the Reaction Conditions for the Photocatalytic Reaction between 1-decene and $BrCH_2CN$

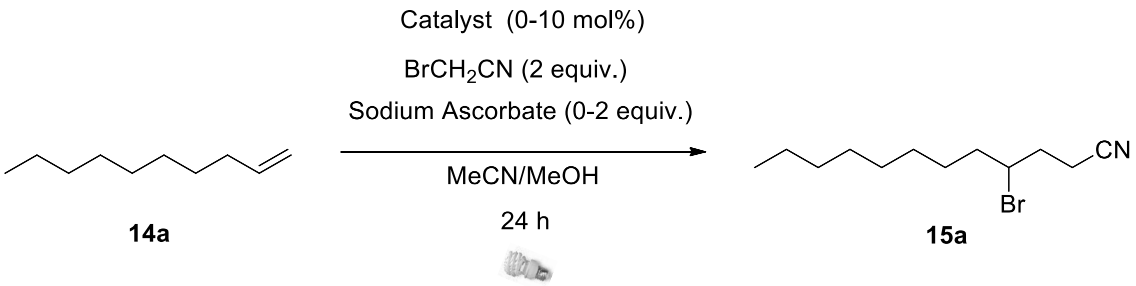

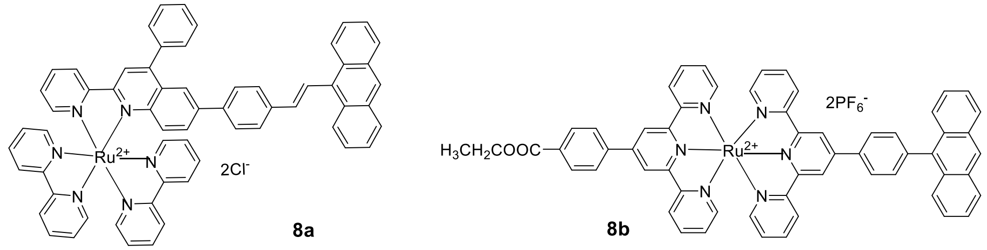

Entry	Catalyst	Catalyst Loading (mol %)	Sodium Ascorbate (equiv.)	Yield (%)[a]
1	Ir(ppy)$_3$ (**8c**)	1	2	98
2	Ru(bpy)$_3$Cl$_2$•6H$_2$O (**8d**)	1	2	78
**3**	**(8a)**	**1**	**2**	**84**
4	(**8b**)	1	2	63
5	(**8a**)	1	-	0
6[b]	(**8a**)	1	2	0
7	-	-	2	0
8	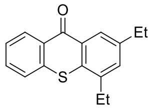 Thioxanthone (**8e**)	10	2	43
9	Eosin Y (**8f**)	10	2	32
10	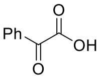 Phenylglyoxylic acid (**8g**)	10	2	33
11	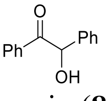 Benzoin (**8h**)	10	2	26
12	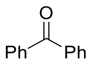 Benzophenone (**8i**)	10	2	0

[a] Isolated yield. The reaction was performed with 1-decene (35 mg, 0.25 mmol), bromoacetonitrile (60 mg, 0.50 mmol), catalyst (0-10 mol%, 0.00-0.025 mmol), sodium ascorbate (0-100 mg, 0-0.50 mmol), acetonitrile (2 mL) and methanol (1.5 mL) under household bulb irradiation for 24 h. [b] The reaction was performed in the dark.

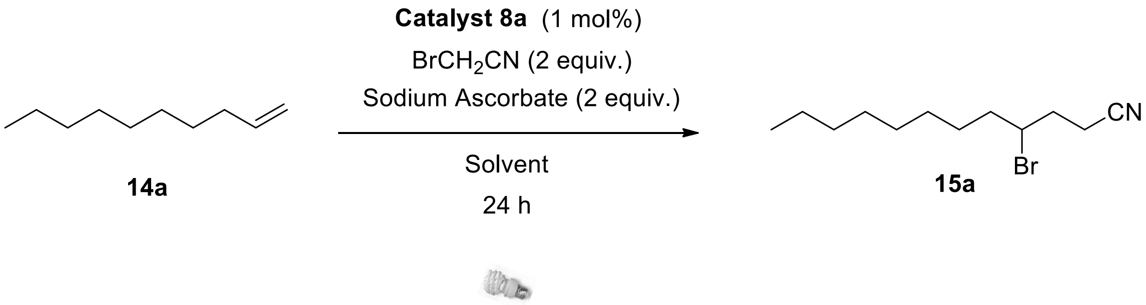

14a 15a

Entry	Solvent	Yield (%)[a]
**1**	**MeCN/MeOH (6:4)**	**84**
2	MeCN	48
3	MeOH	28
4	DMF	80
5	DMSO	0
6	CH_2Cl_2	66

[a] Isolated yield. The reaction was performed with 1-decene (35 mg, 0.25 mmol), bromoacetonitrile (60 mg, 0.50 mmol), catalyst (1 mol%, 0.0025 mmol), sodium ascorbate (100 mg, 0.50 mmol), and solvent (3.5 mL), under household bulb irradiation for 24 h.

Synthesis of the Photocatalysts

Dichlorobis-(2,2´-bipyridine)-ruthenium(II) dihydrate (7)[1]

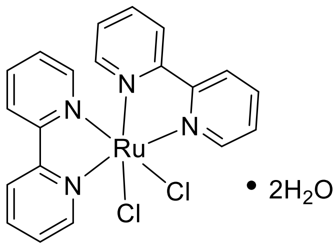

A flame-dried flask was charged with ruthenium trichloride (15.60 g, 75.20 mmol) and 2,2'-bipyridine **6** (18.70 g, 120.00 mmol) in *N,N*-dimethylformamide (600 mL) and heated to reflux for 3 h. Most of the solvent was distilled off and the remaining solution was cooled to room temperature, diluted with acetone (500 mL) and kept overnight at 0 ºC. The crystals formed were filtrated and washed with water. The crude product was suspended in water-ethanol (1:1) (2500 mL) and heated to reflux for 1 h, filtered from the insoluble solid and treated carefully with lithium chloride (300 g, 7.00 mol). Ethanol was distilled off and the resulting water solution was cooled in an ice bath. Dark crystals were precipitated and collected through filtration. (62% yield). **^{1}H NMR** (500 MHz, DMSO-d6) δ: 9.97 (2H, dd, J = 5.6 and 1.1 Hz, ArH), 8.64 (2H, d, J = 8.2 Hz, ArH), 8.48 (2H, d, J = 8.2 Hz, ArH), 8.07 (2H, dt, J = 7.8 and 1.5 Hz, ArH), 7.77 (2H, dt, J = 6.3 and 1.3 Hz, ArH), 7.68 (2H, dt, J = 7.8 and 1.5 Hz, ArH), 7.51 (2H, d, J = 5.6 Hz, ArH), 7.10 (2H, dt, J = 6.6 and 1.3Hz, ArH); **^{13}C NMR** (125 MHz, $CDCl_3$) δ: 160.2, 158.2, 153.2, 151.9, 134.5, 133.3, 125.3, 125.2, 122.8, 122.5.

4-Phenyl-2-(pyridin-2-yl)-6-(4-vinylphenyl)quinolone[2]

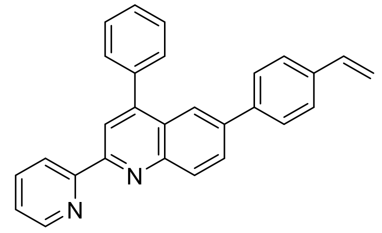

A degassed three-necked flask equipped with a reflux condenser and a magnetic stirrer, was charged with 6-bromo-4-phenyl-2-(pyridin-2-yl)quinolone **1** (4.00 g, 11.00 mmol), (4-vinylphenyl)boronic acid **2** (4.50 g, 22.00 mmol) and $Pd(PPh_3)_4$ (0.63 g, 0.55 mmol). Then, degassed toluene (200 mL) and a degassed 2 M aqueous solution of Na_2CO_3 (50 mL) were added. The reaction was heated to reflux and left stirring vigorously for 48 h. After cooling to room temperature, the resulting mixture was filtered and the organic layer was separated, washed with H_2O (3 x 50 mL), and evaporated under reduced pressure. The obtained solid was suspended in methanol and filtered to afford a pale white solid. Drying under vacuum resulted in the final product (3.40 g, 80%). All data matched literature data.

{6-{4-[2-(Anthracen-9-yl)vinyl]phenyl}-4-phenyl-2-(pyridin-2-yl) quinoline} (LPI) (4)[2]

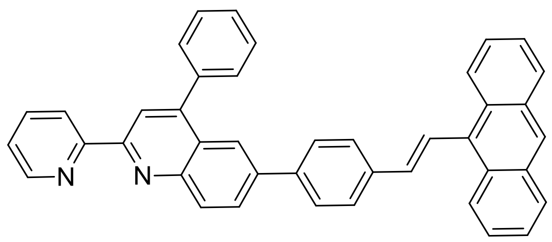

A degassed three-necked flask equipped with a reflux condenser and a magnetic stirrer, was charged with 4-phenyl-2-(pyridin-2-yl)-6-(4-vinylphenyl)quinolone (1.50 g, 3.90 mmol), 9-bromoanthracene **3** (1.51 g, 5.87 mmol), $Pd(OAc)_2$ (18 mg, 71.3 μmol), and tris(3-methylphenyl)phosphine (72 mg, 235 μmol) under argon

atmosphere. Then, DMF (45 mL) along with Et_3N (6 mL) were added and the reaction mixture was carefully degassed again. The reaction mixture was heated to reflux for 72 h and after cooling to room temperature was poured into a mixture of crushed ice and water (500 mL). A yellowish solid started to form immediately. This mixture was filtered and repeatedly washed with water, in order to remove any excess of solvents. The reaction mixture was stirred vigorously for 24 h in ethanol and filtered again. After drying under reduced pressure at 50 °C for 24 h, the reaction mixture was redissolved in the minimum amount of DMF and precipitated with H_2O to afford ligand **LP1 4** as a yellow powder (1.31 g, 60%). All data matched literature data.

[Ru(LP1)(bpy)₂][Cl₂]₂((8a)[3]

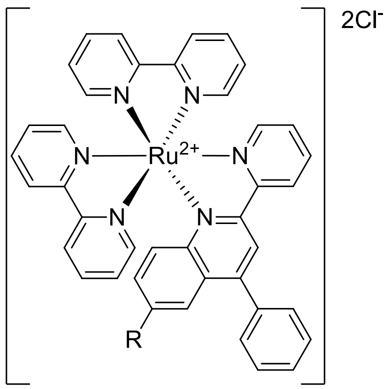

A flame-dried 10 mL flask equipped with a reflux condenser and a magnetic stirrer, was charged with **LP1 4** (83 mg, 0.15 mmol), dichlorobis(2,2'-bipyridine)ruthenium (II) dihydrate **7** (100 mg, 0.19 mmol) and ethylene glycol (4 mL). The reaction mixture was heated at 215 °C for 4 h and then cooled to room temperature. The solvent was subsequently removed by direct distillation under high vacuum. The deep red residual solid was dissolved in ethanol and filtered. The filtrate was then concentrated *in vacuo* and the remaining solid was transferred to a G4 sintered glass crucible using diethyl ether and washed repeatedly with toluene and diethyl ether. Subsequent drying under high vacuum at 60 °C afforded $[Ru(LP1)(bpy)_2]^{2+}$ $2Cl^-$ (**8a**) (0.12 g, 90% yield). All data matched literature data.

Ethyl 4-([2,2':6',2''-terpyridin]-4'-yl)benzoate[4]

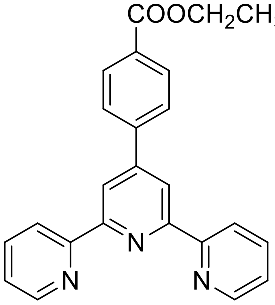

A round-bottom flask flask equipped with a magnetic stirrer, was charged with 2-acetylpyridine **9** (290 mg, 2.40 mmol) and 4-carboxybenzaldehyde **10** (180 mg, 1.20 mmol) in methanol (8 mL). Then, 15% aq. KOH (7.2 mL) and saturated aq. NH_4OH (0.8 mL) were added and the reaction mixture was left stirring at room temperature for 3 days. The emulsion formed was filtered and the solid was washed with $CHCl_3$ (4 mL) and cooled CH_3OH/H_2O (1:1, 4 mL). The crude product was suspended in CH_3OH/H_2O (80:20) and stirred at 35 °C, until a clear solution was obtained. Then, the reaction mixture was acidified to pH = 2 by the addition of 1M HCl, resulting in the formation of a white precipitate, that was collected by filtration and rinsed with cold water. The solid was dried to give a white solid (340 mg, 80% yield). All data matched literature data.

The solid (340 mg, 1.00 mmol) was added to a round-bottom flask equipped with a condenser and a magnetic stirrer and was dissolved in ethanol (10 mL). Catalytic amount of sulfuric acid was added and the reaction mixture was heated to reflux for 3 days. Then, the reaction mixture was concentrated *in vacuo* and the crude oil was extracted with CH_2Cl_2 (3 x 30 mL). The pure product was isolated as white flakes (260 mg, 70%). All data matched literature data.

$RuCl_3$(tpy–C_6H_4–COOEt) (11)[5]

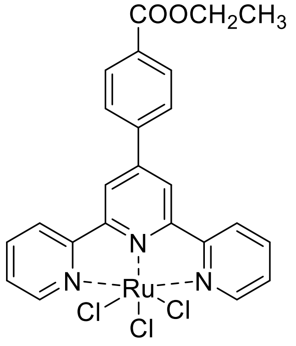

A flame-dried flask equipped with a magnetic stirrer was charged with $RuCl_3$x3H_2O (288 mg, 1.10 mmol), ethyl 4-([2,2':6',2''-terpyridin]-4'-yl)benzoate (381 mg, 1.00 mmol) and dry methanol (20 mL). The reaction mixture was heated to reflux for 2 h. After completion, the reaction mixture was left to cool to room temperature. Product **11** was obtained through filtration and washed thoroughly with absolute ethanol (20 mL) (501 mg, 80% yield).

4'-(4-(Anthracen-9-yl)phenyl)-2,2':6',2''-terpyridine (13)[6]

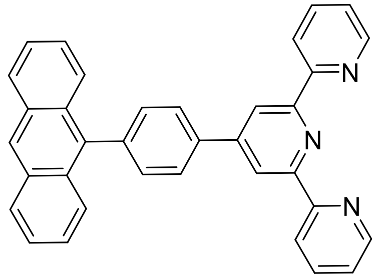

A round-bottom flask equipped with a magnetic stirrer was charged with 4-formyl phenyl boronic acid **12** (0.50 g, 3.33 mmol), 2-acetylpyridine **9** (1.34 g, 11.10 mmol), NaOH (0.29 g, 7.33 mmol) and EtOH (20 mL). The reaction mixture was stirred at room temperature for 4 h. Then, concentrated aqueous NH_3 solution (10 mL) was added and the suspension was stirred at 65 °C for 12 h. The precipitate was filtered and washed with water and isopropanol to afford the product as a white solid (0.85 mg, 72% yield). All data matched literature data.

A three-necked round-bottom flask was charged with (4-([2,2':6',2"-terpyridin]-4'-yl)phenyl)boronic acid (0.83 g, 2.34 mmol), 9-bromoanthracene **3** (0.30 g, 1.17 mmol), Na_2CO_3 (0.74 g, 7.02 mmol), toluene (26 mL) and methanol (37 mL) and the reaction mixture was degassed via three freeze-pump-thaw cycles. Then, $Pd(PPh_3)_4$ (0.20 g, 0.18 mmol) was added under argon atmosphere. The mixture was heated to reflux for 48 h. Then, the reaction mixture was concentrated *in vacuo* and $CHCl_3$ (50 mL) was added and washed with H_2O. The organic layer was dried over anhydrous Na_2SO_4, filtered and concentrated. The residue was purified by column chromatography on alumina with CH_2Cl_2/CH_3OH (100:1) as eluent to give **13** as a white solid (0.50 g, 88% yield). All data matched literature data.

Ru[(tpy-C_6H_4-anthracene)(tpy-C_6H_4-COOEt)][PF_6]$^-_2$ (8b)

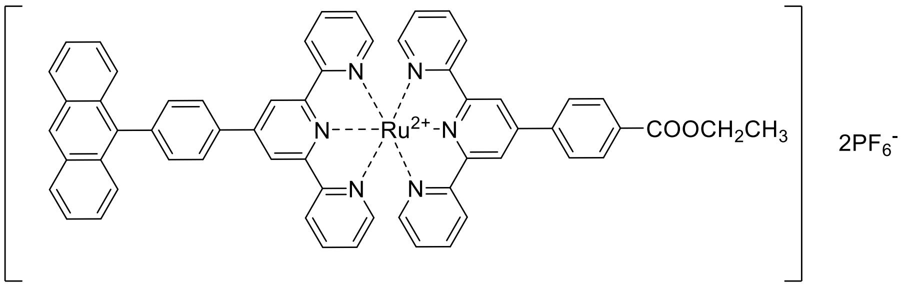

A flame-dried flask equipped with a reflux condenser and a magnetic stirrer was charged with **11** (0.10 g, 0.16 mmol) and 4'-(4-(Anthracen-9-yl)phenyl)-2,2':6',2"-terpyridine **13** (0.58 mg, 0.12 mmol). Then, ethanol (10 mL), CH_2Cl_2 (20 mL) and *N*-methylmorpholine (0.30 mL) were added and the reaction mixture was heated to reflux for 3 days. The red reaction mixture was cooled to room temperature, filtered through Celite and washed with tetrahydrofuran. The pad of Celite was washed with tetrahydrofuran (50 mL), CH_2Cl_2 (50 mL) and acetonitrile (50 mL). The filtrate was concentrated *in vacuo* and methanol (20 mL) was added to the residue. Then, aqueous solution of ammonium hexafluorophosphate (1.50 g, 200 mL) was added and the reaction mixture was cooled at 4 °C for 24 h. The precipitate was obtained by filtration and washed with ice-cold water (100 mL) and diethyl ether (50 mL) to afford the

product **8b** as a brown solid; 85% yield; **^{1}H NMR** (500 MHz, DMSO-d6) *δ*: 9.68 (2H, d, *J* = 6.6 Hz, ArH), 9.57 (2H, d, *J* = 6.6 Hz, ArH), 9.25-9.10 (4H, m, ArH), 9.06 (1H, s, ArH), 8.90 (1H, s, ArH), 8.84-8.53 (5H, m, ArH), 8.43-8.00 (10H, m, ArH), 7.92-7.47 (10H, m, ArH), 7.33 (2H, m, ArH), 4.43 (2H, q, *J* = 7.0 Hz, OCH_2), 1.49-1.33 (3H, t, *J* = 7.0 Hz, CH_3); **^{13}C NMR** (125 MHz, DMSO-d6) (Based on HSQC signals, due to low solubility of the complex, it was not possible to obtain a good ^{13}C-NMR)[5] *δ*: 152.2, 138.1, 137.7, 132.0, 129.9, 128.7, 128.0, 127.7, 127.6, 127.0, 124.9, 121.3, 125.8, 125.5, 124.0, 121.4, 119.9, 118.1, 60.9, 13.9.

Synthesis of Starting Materials

(*E*)-Octadec-9-ene (14e)[7]

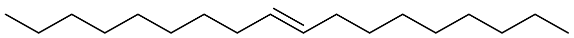

A flame-dried 20 mL flask was charged Grubbs catalyst 2nd generation (3% mol, 255 mg, 0.30 mmol) and the flask was degassed. Then, dry CH_2Cl_2 (5 mL) and 1-decene (1.9 mL, 10.00 mmol) were added and the reaction mixture was left at r.t. overnight. The solvent was concentrated *in vacuo*. The crude material was purified by column chromatography on silica gel (Pet. Ether); Colorless oil; 50% yield; **^{1}H NMR** (200 MHz, $CDCl_3$) δ: 5.44-5.34 (2H, m, 2 x =CH), 2.05-1.94 (4H, m, 2 x CH_2), 1.34-1.25 (24H, m, 12 x CH_2), 0.89 (6H, t, J = 6.3 Hz, 2 x CH_3); **^{13}C NMR** (50 MHz, $CDCl_3$) δ: 130.5, 32.8, 32.1, 29.9, 29.7, 29.5, 29.4, 22.9, 14.3; **MS** 253 $[M+H]^+$.

(2-(Allyloxy)ethyl)cyclohexane (14i)[8]

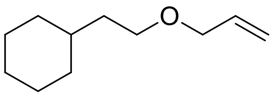

A flame-dried 50 mL flask was charged with 2-cyclohexylethanol (696 mg, 5.00 mmol) in dry THF (10 mL). The mixture was cooled at 0 °C and sodium hydride (60 wt% on mineral oil, 300 mg, 7.50 mmol) was added in one portion. After stirring for 10 min, allyl bromide (0.65 mL, 7.50 mmol) was added dropwise at the same temperature. The cooling bath was removed and the suspension was stirred at room temperature for 30 min. The reaction mixture was then heated to reflux for 16 h. After full conversion, the suspension was cooled to room temperature and quenched by the addition of a saturated aqueous solution of NH_4Cl (10 mL). The biphasic mixture was then extracted with Et_2O (3 x 10 mL). The combined organic layers were dried over $NaSO_4$ and concentrated *in vacuo*. The crude material was purified by column chromatography on silica gel (Pet. Ether/EtOAc 100:1); Colorless oil; 87% yield; **^{1}H**

NMR (200 MHz, $CDCl_3$) *δ*: 5.98-5.73 (1H, m, =CH), 5.21 (1H, d, *J* = 17.2 Hz, =CH*H*), 5.10 (1H, d, *J* = 10.4 Hz, =CH*H*), 3.90 (2H, d, *J* = 4.0 Hz, OCH_2), 3.40 (2H, t, *J* = 6.6 Hz, OCH_2), 1.76-1.49 (5H, m, 2 x CH_2 and CH), 1.49-1.33 (3H, m, 3 x CH*H*), 1.22-1.05 (3H, m, 3 x CH*H*), 0.73-0.69 (2H, m, 2 x CH*H*); **^{13}C NMR** (50 MHz, $CDCl_3$) *δ*: 135.0, 116.4, 71.7, 68.2, 37.1, 34.4, 33.3, 26.5, 26.2; **MS** 169 $[M+H]^+$.

(4-(Allyloxy)butyl)benzene (14j)[9]

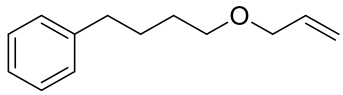

A flame-dried 50 mL flask was charged with 4-phenylbutan-1-ol (300 mg, 2.00 mmol) and dry THF (4 mL). The mixture was cooled at 0 °C and sodium hydride (60 wt% on mineral oil, 120 mg, 3.00 mmol) was added in one portion. After stirring for 10 min, allyl bromide (0.26 mL, 3.00 mmol) was added dropwise at the same temperature. The cooling bath was removed and the suspension was stirred at room temperature for 30 min. The reaction mixture was then heated to reflux for 16 h. After full conversion, the suspension was cooled to room temperature and quenched by the addition of a saturated aqueous solution of NH_4Cl (10 mL). The biphasic mixture was then extracted with Et_2O (3 x 10 mL). The combined organic layers were dried over $NaSO_4$ and concentrated *in vacuo*. The crude material was purified by column chromatography on silica gel (Pet. Ether/EtOAc 100:1); Colorless oil; 87% yield; **^{1}H NMR** (200 MHz, $CDCl_3$) *δ*: 7.41-7.12 (5H, m, ArH), 6.09-5.86 (1H, m, =CH), 5.33 (1H, d, *J* = 17.2 Hz, =CH*H*), 5.23 (1H, d, *J* = 10.4 Hz, =CH*H*), 4.02 (2H, d, *J* = 7.2 Hz, OCH_2), 3.51 (2H, t, *J* = 5.8 Hz, OCH_2), 2.70 (2H, t, *J* = 7.2 Hz, CH_2), 1.88-1.59 (4H, m, 2 x CH_2); **^{13}C NMR** (50 MHz, $CDCl_3$) *δ*: 142.3, 134.9, 128.3, 128.1, 125.6, 116.6, 71.7, 70.1, 35.6, 29.3, 28.0; **MS** 191 $[M+H]^+$.

Allyl benzoate (14k)[10]

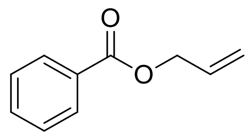

A 50 mL flask was charged with benzoic acid (366 mg, 3.00 mmol) in dry CH_2Cl_2 (28 mL). To the stirred solution was then added *N,N'*-dicyclohexylcarbodiimide (738 mg, 3.60 mmol), 4-dimethylaminopyridine (248 mg, 2.01 mmol) and allyl alcohol (174 mg, 3.00 mmol) and the reaction mixture was left stirring for 24 h. The precipitate was removed by filtration and the solvent was removed *in vacuo*. The crude material was purified by column chromatography on silica gel (Pet. Ether/EtOAc 100:1); Colorless oil; 83% yield; **^{1}H NMR** (200 MHz, $CDCl_3$) δ: 8.16-8.00 (2H, m, ArH), 7.61-7.35 (3H, m, ArH), 6.16-5.93 (1H, m, =CH), 5.48-5.23 (2H, m, $=CH_2$), 4.86-4.77 (2H, m, OCH_2); **^{13}C NMR** (50 MHz, $CDCl_3$) δ: 166.0, 132.8, 130.0, 129.5, 128.2, 118.0, 65.4; **MS** 163 $[M+H]^+$.

N-Allylbenzamide (14l)[11]

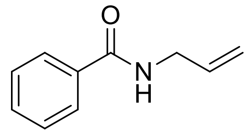

To a 50 mL round bottom flask, allyl-amine (213 mg, 3.00 mmol) was dissolved in dry CH_2Cl_2 (30 mL). The solution is cooled to 0 ^{o}C and then DIPEA (1.55 mL, 9.00 mmol), WSCI (864 mg, 4.50 mmol), HOBt (405 mg, 3.00 mmol) and benzoic acid (366 mg, 3.00 mmol) were added and the reaction mixture was left 1 h in 0 ^{o}C and to r.t. overnight. Then the solvent was evaporated, the mixture was diluted with EtOAc (30 mL) and the organic layer was washed with H_2O (1 x 20 mL), HCl 1 N (1 x 20 mL), $NaHCO_3$ (1 x 20 mL) and brine (1 x 20 mL). The organic layer was dried over $NaSO_4$ and concentrated *in vacuo*. The crude material was purified by column chromatography on silica gel (Pet. Ether/EtOAc 8:2); Colorless oil; 83% yield; **^{1}H**

NMR (200 MHz, $CDCl_3$) *δ*: 7.80-7.46 (2H, m, ArH), 7.42-7.41 (1H, m, ArH), 7.40-7.38 (2H, m, ArH), 6.49 (1H, br s, NH), 5.96-5.87 (1H, m, =CH), 5.26-5.14 (2H, m, $=CH_2$), 4.08-4.04 (2H, m, CH_2); **^{13}C NMR** (50 MHz, $CDCl_3$) *δ*: 167.5, 134.6, 134.3, 131.6, 128.6, 127.1, 116.7, 42.5; **MS** 162 $[M+H]^+$.

(4-Methylenecyclohexyl)benzene (14o)[12]

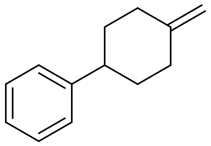

A flame-dried 50 mL flask was charged with methyltriphenylphosphonium bromide (2.30 g, 6.50 mmol) in dry THF (20 mL) and the flask was degassed. Then, the solution was cooled to 0 ^{o}C, sodium *tert*-butoxide (480 mg, 5.00 mmol) and sodium bis(trimethylsilyl)amide (1M in THF) (5 mL, 5.00 mmol) were added and the reaction mixture was left on ice-bath for 0.5 h. Then, 4-phenylcyclohexanone (870 mg, 5.00 mmol) dissolved in dry THF (5 mL) was added dropwise. The reaction mixture was left at r.t. overnight. The reaction mixture was quenched with HCl 1N (5 mL) at 0 ^{o}C and extracted with ethyl acetate (3 x 10 mL). The combined organic layers were treated with brine (1 x 20 mL), dried over $NaSO_4$ and concentrated *in vacuo*. The crude material was purified by column chromatography on silica gel (Pet. Eth.); Colorless oil; 77% yield; **^{1}H NMR** (200 MHz, $CDCl_3$) *δ*: 7.47-7.28 (5H, m, ArH), 4.85 (1H, d, *J* = 1.4 Hz, =CH*H*), 4.84 (1H, d, *J* = 1.4 Hz, =CH*H*), 2.87-2.75 (1H, m, CH), 2.61-2.54 (2H, m, 2 x CHH), 2.39-2.26 (2H, m, 2 x CHH), 2.16-2.10 (2H, m, 2 x CHH), 1.80-1.60 (2H, m, 2 x CHH); **^{13}C NMR** (50 MHz, $CDCl_3$) *δ*: 148.6, 146.7, 128.3, 126.7, 125.9, 107.4, 44.0, 35.4, 35.0; **MS** 173 $[M+H]^+$.

N-(But-3-en-1-yl)benzamide (14t)[13]

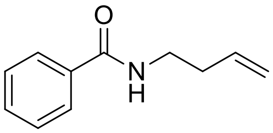

A 50 mL flask was charged with but-3-en-1-amine (142 mg, 2.00 mmol) dissolved in dry CH_2Cl_2 (21 mL). The reaction mixture was cooled to 0 °C and *N*,*N*-diisopropylethylamine (1.03 mL, 6.00 mmol), WSCI (576 mg, 3.00 mmol), *N*-hydroxybenzotriazole (270 mg, 2.00 mmol) and benzoic acid (244 mg, 2.00 mmol) were added. The mixture was cooled to 0 °C for 1 h. The stirred solution was then left at room temperature for 24 h. The solvent was removed *in vacuo*. The obtained crude mixture was dissolved in EtOAc (20 mL) and washed with H_2O (10 mL), HCl (1N) (10 mL), $NaHCO_3$ (10 mL) and H_2O (10 mL). The organic layer was dried over $NaSO_4$ and concentrated *in vacuo.* The crude material was purified by column chromatography on silica gel (Pet. Ether/EtOAc 100:1); Colorless oil; 70% yield; **^{1}H NMR** (200 MHz, $CDCl_3$) δ: 7.81-7.65 (2H, m, ArH), 7.42-7.13 (3H, m, ArH), 5.85-5.58 (1H, m, =CH), 5.11-4.90 (2H, m, =CH_2), 3.41 (2H, m, NCH_2), 2.28 (2H, q, J = 6.7 Hz, CH_2); **^{13}C NMR** (50 MHz, $CDCl_3$) δ: 167.5, 135.1, 134.4, 130.9, 128.1, 126.7, 116.6, 38.8, 33.5; **MS** 176 $[M+H]^+$.

Pent-4-en-1-yl benzoate (14u)[13]

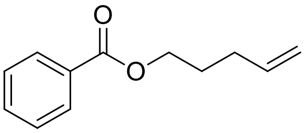

A 50 mL flask was charged with benzoic acid (366 mg, 3.00 mmol) dissolved in dry CH_2Cl_2 (28 mL). To the stirred solution was then added *N*,*N'*-dicyclohexylcarbodiimide (738 mg, 3.60 mmol), 4-dimethylaminopyridine (248 mg,

2.01 mmol) and 4-penten-1-ol (258 mg, 3.00 mmol), and the reaction mixture was left stirring for 24 h. The precipitate was removed by filtration and the solvent was removed *in vacuo*. The crude material was purified by column chromatography on silica gel (Pet. Ether/EtOAc 100:1); Colorless oil; 90% yield; **^{1}H NMR** (200 MHz, $CDCl_3$) δ: 8.10-7.98 (2H, m, ArH), 7.59-7.34 (3H, m, ArH), 5.97-5.72 (1H, m, =CH), 5.13-5.05 (2H, m, $=CH_2$), 4.33 (2H, t, J = 6.5 Hz, OCH_2), 2.22 (2H, m, CH_2), 1.94-1.77 (2H, m, CH_2); **^{13}C NMR** (50 MHz, $CDCl_3$) δ: 166.0, 137.1, 132.4, 130.1, 129.2, 128.0, 115.0, 63.9, 29.8, 27.6; **MS** 191 $[M+H]^{+}$.

General Procedure for the Photocatalytic Reaction between Olefins and $BrCH_2CN$

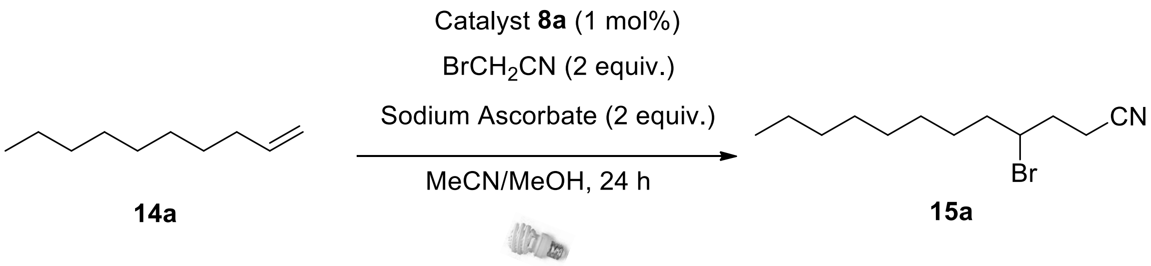

In a glass vial with a screw cap containing $[Ru(LP1)(bpy)_2]^{2+}2Cl^-$ (**8a**) (1.5 mg, 2.5 x 10^{-3} mmol) in acetonitrile (2 mL) and methanol (1.5 mL), alkene (0.25 mmol), $BrCH_2CN$ (60 mg, 0.50 mmol) and sodium ascorbate (100 mg, 0.50 mmol) were added consecutively. The vial was sealed with a screw cap and left stirring under household bulb irradiation (2 x 80W household lamps, see photos below) for 24 h. The desired product was isolated after purification by column chromatography.

A

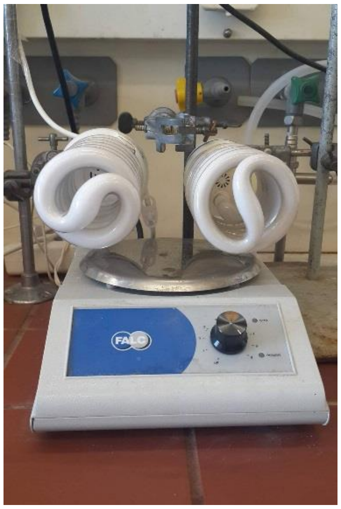

B

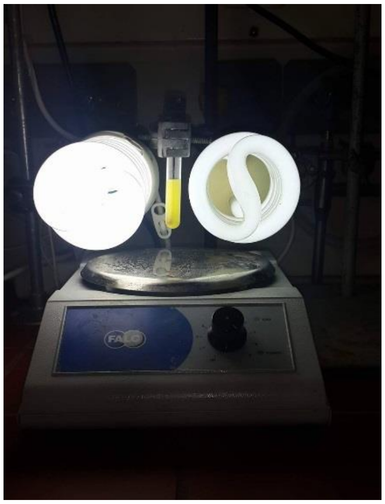

Scheme. **A**: 2 x 80W fluorescent household lamps utilized for the photocatalytic reaction. Bulbs are placed symmetrically 3 cm away from the reaction tube. **B**: Beginning of the reaction.

4-Bromododecanenitrile (15a)[14]

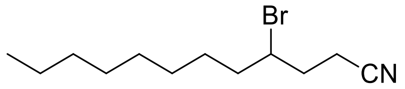

Yellow oil; 84% yield; **^{1}H NMR** (200 MHz, $CDCl_3$) *δ*: 4.13-3.95 (1H, m, CHBr), 2.60 (2H, t, *J* = 6.9 Hz, $NCCH_2$), 2.22-1.98 (2H, m, CH_2), 1.90-1.73 (2H, m, CH_2), 1.61-1.39 (2H, m, CH_2), 1.37-1.12 (10H, m, 5 x CH_2), 0.87 (3H, t, *J* = 6.2 Hz, CH_3); **^{13}C NMR** (50 MHz, $CDCl_3$) *δ*: 118.8, 55.0, 38.8, 34.5, 31.7, 29.3, 29.1, 28.8, 27.4, 22.6, 16.0, 14.0; **MS** 260 $[M+H]^+$.

4-Bromooctanenitrile (15b)[14]

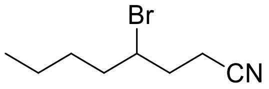

Yellow oil; 99% yield; **^{1}H NMR** (200 MHz, $CDCl_3$) *δ*: 4.15-3.96 (1H, m, CHBr), 2.61 (1H, t, *J* = 6.7 Hz, $NCCH_2$), 2.23-1.99 (2H, m, CH_2), 1.93-1.75 (2H, m, CH_2), 1.60-1.22 (4H, m, 2 x CH_2), 0.92 (3H, t, *J* = 7.0 Hz, CH_3); **^{13}C NMR** (50 MHz, $CDCl_3$) *δ*: 118.8, 55.0, 38.6, 34.5, 29.5, 22.0, 16.0, 13.9; **MS** 204 $[M+H]^+$.

2-(2-Bromocyclooctyl)acetonitrile (15c)[14]

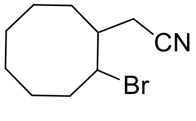

Pale yellow oil; 57% yield; Mixture of diastereomers (77:23); **^{1}H NMR** (200 MHz, $CDCl_3$) *δ*: 4.51-4.26 (0.77H, m, CHBr), 4.22-4.08 (0.23H, m, CHBr), 2.40-2.17 (3H, m, $CNCH_2$ and CH), 2.15-1.19 (12H, m, 6 x CH_2); **^{13}C NMR** (50 MHz, $CDCl_3$) *δ*: 118.9,

118.9, 56.2, 55.8, 35.6, 35.5, 35.1, 34.3, 33.7, 32.8, 30.3, 30.2, 30.1, 29.4, 25.8, 25.5, 25.4, 24.9, 24.6, 24.2; **MS** 230 $[M+H]^+$.

trans-2-(-3-Bromobicyclo[2.2.1]heptan-2-yl)acetonitrile (15d)[14]

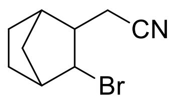

Yellow oil; 77% yield; Mixture of diastereomers (80:20); **^{1}H NMR** (200 MHz, $CDCl_3$) δ: 4.18 (0.80 H, dd, J = 7.3 and 1.8 Hz, CHBr), 3.81-3.73 (0.20H, m, CHBr), 2.61-2.26 (3H, m, $CNCH_2$ and CH), 2.19-2.05 (1H, m, CH), 1.89-1.77 (1H, m, CH), 1.70-1.43 (3H, m, 3 x CH*H*), 1.39-1.17 (3H, m, 3 x CH*H*); **^{13}C NMR** (50 MHz, $CDCl_3$) δ: 119.6, 58.6, 57.3, 49.7, 48.1, 44.5, 44.2, 42.1, 41.2, 34.7, 33.1, 29.6, 29.1, 27.2, 23.8, 23.1, 21.3; **MS** 214 $[M+H]^+$.

4-Bromo-3-octyldodecanenitrile (15e)

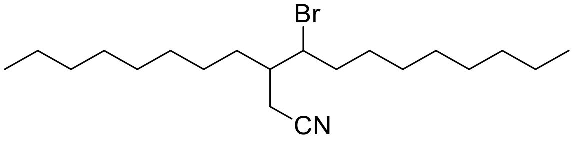

Colorless oil; 72% yield; Mixture of diastereomers (50:50); **^{1}H NMR** (200 MHz, $CDCl_3$) δ: 4.29-4.21 (0.5H, m, CHBr), 4.17-4.08 (0.5H, m, CHBr), 2.63-2.30 (2H, m, 2 x CH*H*CN), 1.97-1.71 (5H, m, 2 x CH_2 and CH), 1.66-1.08 (24H, m, 12 x CH_2), 0.87 (6H, t, J = 6.3 Hz, 2 x CH_3); **^{13}C NMR** (50 MHz, $CDCl_3$) δ: 118.9, 118.7, 60.8, 60.1, 42.0, 41.5, 37.0, 35.7, 32.4, 31.8, 31.7, 29.6, 29.4, 29.2, 29.1, 28.9, 28.1, 27.9, 27.0, 26.5, 22.6, 20.7, 19.6, 14.1; **HRMS** exact mass calculated for $C_{20}H_{38}BrNNa$ $[M+Na]^+$ 394.2080; found: 394.2090.

4-Bromo-5-cyclopentylpentanenitrile (15f)[14]

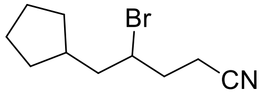

Brown oil; 69% yield; **^{1}H NMR** (200 MHz, $CDCl_3$) δ: 4.15-3.96 (1H, m, CHBr), 2.62 (2H, t, J = 6.7 Hz, $NCCH_2$), 2.22-1.95 (3H, m, CH_2 and CH), 1.92-1.70 (3H, m, 3 x CH*H*), 1.68-1.41 (5H, m, 5 x CH*H*), 1.19-0.95 (2H, m, 2 x CH*H*); **^{13}C NMR** (50 MHz, $CDCl_3$) δ: 118.8, 54.4, 45.2, 38.1, 34.7, 32.5, 31.8, 24.9, 24.8, 15.9; **MS** 230 $[M+H]^+$.

4-Bromo-4-cyclohexylbutanenitrile (15g)[14]

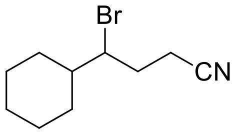

Yellow oil; 55% yield; **^{1}H NMR** (200 MHz, $CDCl_3$) δ: 4.09-3.92 (1H, m, CHBr), 2.72-2.52 (2H, m, $NCCH_2$), 2.19-2.02 (2H, m, CH_2), 1.88-1.47 (6H, m, CH and 5 x CH*H*), 1.36-1.04 (5H, m, 5 x CH*H*); **^{13}C NMR** (50 MHz, $CDCl_3$) δ: 118.8, 62.0, 44.4, 31.8, 30.6, 29.3, 26.0, 25.9, 25.8, 16.4; **MS** 230 $[M+H]^+$.

4-Bromo-6-phenylhexanenitrile (15h)[14]

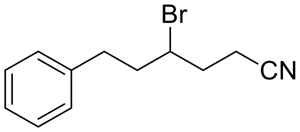

Pale yellow oil; 60% yield; **^{1}H NMR** (200 MHz, $CDCl_3$) δ: 7.40-7.12 (5H, m, ArH), 4.09-3.90 (1H, m, CHBr), 3.00-2.71 (2H, m, $NCCH_2$), 2.67-2.54 (2H,

m, CH_2), 2.22-2.07 (4H, m, 2 x CH_2); **^{13}C NMR** (50 MHz, $CDCl_3$) δ: 140.1, 128.6, 128.4, 126.3, 118.7, 54.1, 40.4, 34.6, 33.5, 15.9; **MS** 252 $[M+H]^+$.

4-Bromo-5-(2-cyclohexylethoxy)pentanenitrile (15i)[14]

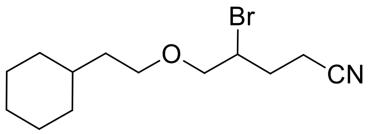

Orange oil; 66% yield; **^{1}H NMR** (200 MHz, $CDCl_3$) δ: 4.20-4.05 (1H, m, CHBr), 3.72 (1H, dd, J = 10.5 and 5.0 Hz, OCH*H*), 3.64-3.36 (3H, m, OCH_2 and OCH*H*), 2.68-2.51 (2H, m, $NCCH_2$), 2.45-2.27 (1H, m, CH*H*), 2.16-1.95 (1H, m, CH*H*), 1.77-1.57 (4H, m, CH and 3 x CH*H*), 1.51-1.34 (3H, m, 3 x CH*H*) 1.27-1.10 (3H, m, 3 x CH*H*), 1.03-0.75 (3H, m, 3 x CH*H*); **^{13}C NMR** (50 MHz, $CDCl_3$) δ: 118.7, 74.1, 69.5, 49.9, 36.9, 34.5, 33.3, 31.2, 26.5, 26.2, 15.7; **MS** 288 $[M+H]^+$.

4-Bromo-5-(4-phenylbutoxy)pentanenitrile (15j)[14]

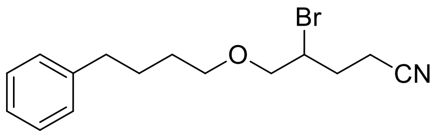

Pale yellow oil; 63% yield; **^{1}H NMR** (200 MHz, $CDCl_3$) δ: 7.34-7.12 (5H, m, ArH), 4.19-4.04 (1H, m, CHBr), 3.72 (1H, dd, J = 11.5 and 6.1 Hz, OCH*H*), 3.59 (1H, dd, J = 11.5 and 6.6 Hz, OCH*H*), 3.49 (2H, t, J = 5.8 Hz, OCH_2), 2.69-2.52 (3H, m, 3 x CH*H*), 2.45-2.25 (2H, m, CH_2), 2.13-1.92 (2H, m, CH_2), 1.73-1.54 (3H, m, 3 x CH*H*); **^{13}C NMR** (50 MHz, $CDCl_3$) δ: 142.2, 128.4, 128.3, 125.7, 118.7, 74.1, 71.3, 49.9, 35.6, 31.2, 29.1, 27.9, 15.7; **MS** 310 $[M+H]^+$.

2-Bromo-4-cyanobutyl benzoate (15k)[14]

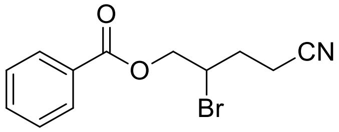

Yellow oil; 43% yield; **^{1}H NMR** (200 MHz, $CDCl_3$) δ: 8.11-7.98 (2H, m, ArH), 7.68-7.40 (3H, m, ArH), 4.65 (1H, dd, *J* = 11.8 and 5.8 Hz, OCH*H*), 4.53 (1H, dd, *J* = 11.8 and 6.3 Hz, OCH*H*), 4.40-4.25 (1H, m, CHBr), 2.74-2.59 (2H, m, $NCCH_2$), 2.45-2.28 (1H, m, CH*H*), 2.24-2.07 (1H, m, CH*H*); **^{13}C NMR** (50 MHz, $CDCl_3$) δ: 165.7, 133.5, 129.7, 129.1, 128.6, 118.3, 67.1, 48.3, 31.1, 15.8; **MS** 282 $[M+H]^+$.

***N*-(2-Bromo-4-cyanobutyl)benzamide (15l)**

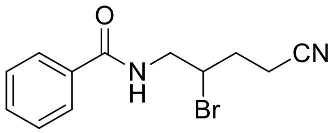

Colorless oil; 55% yield; **^{1}H NMR** (200 MHz, $CDCl_3$) δ: 7.90 (2H, dd, *J* = 8.1 and 1.6 Hz, ArH), 7.74 (1H, d, *J* = 8.0 Hz, NH), 7.50-7.33 (3H, m, ArH), 4.86-4.71 (1H, m, CHBr), 4.17 (1H, dd, *J* = 14.8 and 9.6 Hz, NCH*H*), 3.66 (1H, dd, *J* = 14.8 and 7.0 Hz, NCH*H*), 2.54 (2H, t, *J* = 7.0 Hz, $CNCH_2$), 1.97 (2H, q, *J* = 7.0 Hz, 2 x CH*H*); **^{13}C NMR** (50 MHz, $CDCl_3$) δ: 163.5, 131.5, 128.3, 128.0, 118.8, 77.5, 59.6, 31.0, 13.7; **HRMS** exact mass calculated for $C_{12}H_{13}BrN_2ONa$ $[M+Na]^+$ 303.0103; found: 303.0109.

4-Bromo-5-hydroxypentanenitrile (15m)[14]

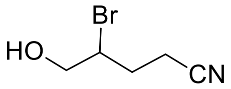

Yellow oil; 71% yield; **^{1}H NMR** (200 MHz, $CDCl_3$) δ: 4.31 (1H, br s, OH), 4.21-4.09 (1H, m, CHBr), 3.88-3.77 (2H, m, OCH_2), 2.68-2.55 (2H, m, $NCCH_2$), 2.37-2.20 (1H, m, CH*H*), 2.16-2.03 (1H, m, CH*H*); **^{13}C NMR** (50 MHz, $CDCl_3$) δ: 118.6, 66.4, 54.8, 30.5, 15.8; **MS** 177 $[M+H]^+$.

4,6-Dibromohexanenitrile (15n)[14]

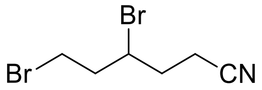

Colorless oil; 57% yield; **^{1}H NMR** (200 MHz, $CDCl_3$) δ: 4.33-4.17 (1H, m, CHBr), 3.66-3.50 (2H, m, CH_2Br), 2.71-2.55 (2H, m, $NCCH_2$), 2.39-2.25 (2H, m, CH_2), 2.23-2.05 (2H, m, CH_2); **^{13}C NMR** (50 MHz, $CDCl_3$) δ: 118.4, 52.0, 41.0, 34.3, 30.4, 16.0; **MS** 253 $[M+H]^+$.

3-(1-Bromo-4-phenylcyclohexyl)propanenitrile (15oA)

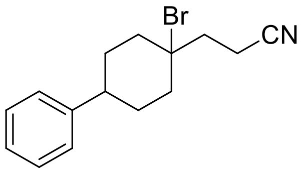

Colorless oil; 25% yield; **^{1}H NMR** (200 MHz, $CDCl_3$) δ: 7.36-7.18 (5H, m, ArH), 2.79-2.66 (2H, m, 2 x C*H*HCN), 2.58-2.41 (1H, m, CH), 2.32-2.15 (3H, m, 3 x CH*H*), 2.10-1.95 (2H, m, 2 x CH*H*), 1.94-1.79 (2H, m, 2 x CH*H*), 1.69-1.49 (3H, m, 3 x CH*H*); **^{13}C NMR** (50 MHz, $CDCl_3$) δ: 146.0, 128.5, 126.8,

126.3, 119.6, 72.2, 43.4, 42.5, 40.7, 30.1, 13.9; **HRMS** exact mass calculated for $C_{15}H_{18}BrNNa$ [M+Na]$^+$ 314.0515; found: 314.0507.

3-(1,2,3,6-Tetrahydro-[1,1'-biphenyl]-4-yl)propanenitrile (15oB)[15]

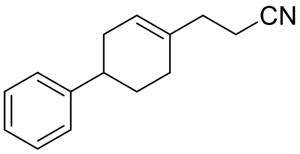

Colorless oil; 50% yield; ^{1}**H NMR** (200 MHz, $CDCl_3$) δ: 7.36-7.15 (5H, m, ArH), 5.64 (1H, s, =CH), 2.86-2.69 (1H, m, CNCH*H*), 2.55-2.44 (2H, m, CNCH*H* and CH), 2.42-2.26 (3H, m, 3 x C*H*H), 2.22-1.97 (4H, m, 4 x CH*H*), 1.89-1.70 (1H, m, CH*H*); ^{13}**C NMR** (50 MHz, $CDCl_3$) δ: 146.6, 133.8, 128.4, 126.8, 126.1, 123.5, 119.6, 39.7, 33.2, 32.9, 29.7, 28.4, 16.0; **MS** 212 [M+H]$^+$.

Products from limonene (15p)

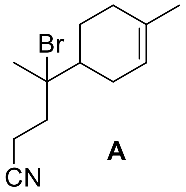

A

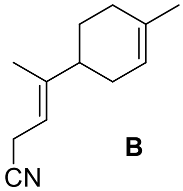

B

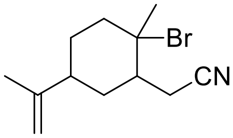

C

Yellow oil; 30% yield; ratio = 50:30:20 **A**:**B**:**C**; ^{1}**H NMR** (200 MHz, $CDCl_3$) δ: 5.44-5.29 [0.8H, m, 0.5H =CH(A) and 0.3H =CH(B)], 5.25-5.13 [0.3H, m, =CH(B)], 4.92 [0.2H, s, =CH(C)], 4.81 [0.2H, s, =CH(C)], 3.04 [0.6H, d, J = 6.8 Hz, CH_2CN (B)], 2.78-2.68 [0.4H, m, CH_2CN(C)], 2.57-2.18 [2H, m,

CH_2CN(A) and CH_2(A)], 2.17-1.85 (3H, m, CH and 2 x CH*H*), 1.84-1.39 (5H, m, CH_3 and 2 x CH*H*), 1.38-1.11 [2.5H, m, 0.5H CH*H*(A), 0.9H CH_3(B), 0.3H CH*H*(B), 0.2H CH*H*(C) and 0.6H CH_3(C)], 0.94-0.81 [1.8H, m, 1.5H CH_3(A) and 0.3H CH*H*(B)], 0.61-0.48 [0.5H, m, CH*H*(A)]; **^{13}C NMR** (50 MHz, $CDCl_3$) δ: 150.2, 146.5, 134.2, 133.9, 133.8, 120.2, 120.0, 119.9, 119.8, 119.5, 119.2, 118.7, 110.3, 109.5, 42.5, 40.8, 39.5, 31.6, 30.7, 30.4, 30.3, 30.1, 29.9, 29.7, 29.6, 29.5, 29.3, 29.0, 28.0, 27.4, 26.9, 26.6, 26.3, 23.8, 23.4, 23.3, 23.2,20.7, 19.1, 17.5, 17.3, 16.4, 16.2, 16.0,14.6, 13.8, 11.0.

Products from beta-pinene (**15q**)

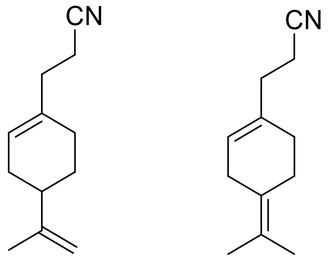

A B

Yellow oil; 35% yield; ratio = 70:30 **A**:**B**; **^{1}H NMR** (200 MHz, $CDCl_3$) δ: 5.61-5.52 [0.7H, m, =CH(A)], 5.42-5.31 [0.3H, m, =CH(B)], 4.78-4.68 [1.4H, m, 2 x =CHH(A)], 2.49-2.39 [1.4H, m, CH_2CN(A)], 2.35-2.25 [1.4H, m, 2 x CHH(A)], 2.21-1.75 [3.9H, m, 2 x C*H*HCN(B), 2 x C*H*H(B), CH(A) and 2 x CHH], 1.74-1.08 [6.1H, m, CH_3(A) and 4 x CHH], 0.87 [1.8H, s, 2 x CH_3(B)], 0.83 [1.8H, s, 2 x CH_3(B)]; **^{13}C NMR** (50 MHz, $CDCl_3$) δ: 149.5, 133.6, 123.4, 123.3, 119.7, 119.6, 108.7, 40.7, 38.7, 38.5, 38.5, 38.4, 32.9, 30.5, 28.3, 27.5, 22.6, 20.7, 16.0.

3-(4-(2-Bromopropan-2-yl)cyclohexylidene)propanenitrile (15qC)

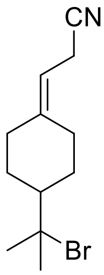

Colourless oil; 30% yield; **^{1}H NMR** (200 MHz, $CDCl_3$) *δ*: 5.60-5.46 (1H, m, =CH), 3.19-3.14 (2H, m, CH_2CN), 2.47-2.36 (2H, m, 2 x C*H*H), 2.33-2.23 (2H, m, 2 x C*H*H), 2.05-1.58 (5H, CH and 4 x C*H*H), 1.08 (6H, s, 2 x CH_3); **^{13}C NMR** (50 MHz, $CDCl_3$) *δ*: 133.8, 123.6, 119.6, 76.4, 48.7, 41.4, 32.8, 28.9, 26.7, 23.6, 22.2, 21.8, 16.0; **HRMS** exact mass calculated for $C_{15}H_{18}BrNNa$ $[M+Na]^+$ 278.0515; found: 278.0517.

1,1,1,2,2,3,3,4,4-Nonafluoro-6-iodotetradecane (15r)[16]

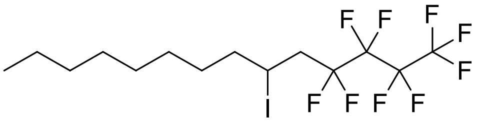

Yellow oil; 70% yield; **^{1}H NMR** (200 MHz, $CDCl_3$) *δ*: 4.41-4.24 (1H, m, CHI), 3.08-2.83 (1H, m, CH*H*CF_2), 2.82-2.60 (1H, m, CH*H*CF_2), 1.86-1.70 (2H, m, CH_2), 1.45-1.16 (12H, m, CH_2), 0.88 (3H, t, *J* = 6.2 Hz, CH_3); **^{13}C NMR** (50 MHz, $CDCl_3$) *δ*: 130-100 (multiple C-F), 41.6 (t, *J* = 20.6 Hz), 40.4-40.3 (m), 31.9, 30.0, 29.4, 29.2, 28.5, 22.7, 20.8, 14.1; **^{19}F NMR** (188 MHz, $CDCl_3$) *δ*: -39.28 (3F), -70.0 (1F, d, *J* = 270 Hz), -73.2 (1F, d, *J* = 270 Hz), -82.8 (2F), -84.15 (2F); **MS** 487 $[M+H]^+$.

5-Octyldihydrofuran-2(3*H*)-one (15s)[11]

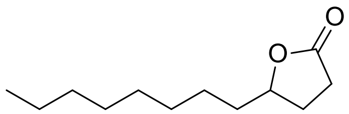

Yellow oil; 47% yield; **^{1}H NMR** (200 MHz, $CDCl_3$) *δ*: 4.53-4.39 (1H, m, OCH), 2.54-2.46 (2H, m, $COCH_2$), 2.37-2.21 (1H, m, CH*H*), 1.93-1.51 (3H, m, 3 x CH*H*), 1.44-1.14 (10H, m, 5 x CH_2), 0.85 (3H, t, *J* = 6.4 Hz, CH_3); **^{13}C NMR** (50 MHz, $CDCl_3$) *δ*: 177.3, 81.0, 35.5, 31.8, 29.3, 29.3, 29.1, 28.8, 27.9, 25.2, 22.6, 14.0; **MS** 199 $[M+H]^+$.

General Procedure for the Photocatalytic Reaction between olefins and $BrCCl_3$

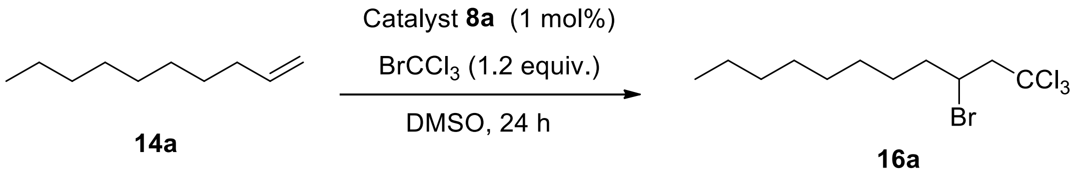

In a glass vial with a screw cap containing $[Ru(LP1)(bpy)_2]^{2+}2Cl^-$ (**8a**) (1.5 mg, 2.5 x 10^{-3} mmol) in DMSO (0.25 mL), alkene (0.25 mmol) and $BrCCl_3$ (100 mg, 0.30 mmol) were added consecutively. The vial was sealed with a screw cap and left stirring under household bulb irradiation (2 x 80W household lamps, see photos below) for 24 h. The desired product was isolated after purification by column chromatography.

A

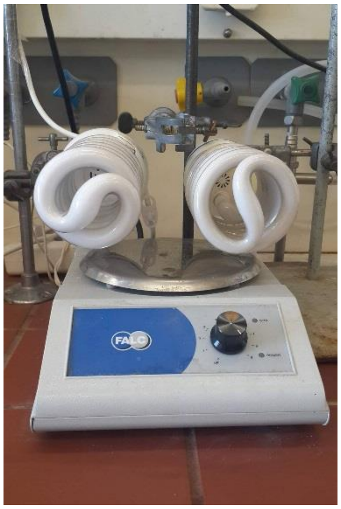

B

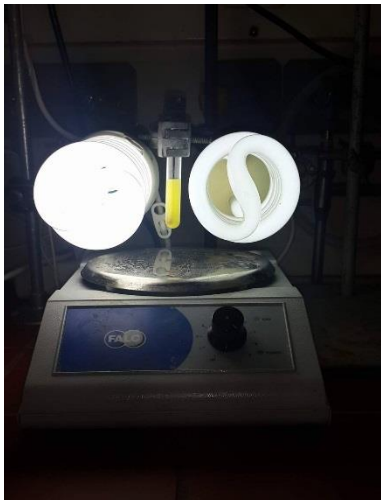

Scheme. **A**: 2 x 80W fluorescent household lamps utilized for the photocatalytic reaction. Bulbs are placed symmetrically 3 cm away from the reaction tube. **B**: Beginning of the reaction.

3-Bromo-1,1,1-trichloroundecane (16a)[17]

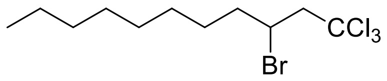

Yellow oil; 90% yield; **^{1}H NMR** (200 MHz, $CDCl_3$) *δ*: 4.32 (1H, m, CHBr), 3.45 (1H, dd, *J* = 15.8 and 5.0 Hz, CCl_3CH*H*), 3.21 (1H, dd, *J* = 15.8 and 5.2 Hz, CCl_3CH*H*), 2.06-1.86 (2H, m, CH_2), 1.62-1.44 (2H, m, CH_2), 1.39-1.19 (10H, m, 5 x CH_2), 0.88 (3H, t, *J* = 6.4 Hz, CH_3); **^{13}C NMR** (50 MHz, $CDCl_3$) *δ*: 97.2, 62.7, 49.2, 39.5, 31.8, 29.4, 29.2, 28.7, 27.2, 22.6, 14.1; **MS** 336 $[M+H]^+$.

3,5-Dibromo-1,1,1-trichloropentane (16b)[18]

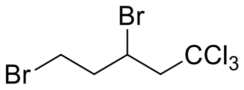

Yellow oil; 64% yield; **^{1}H NMR** (200 MHz, $CDCl_3$) *δ*: 4.64-4.42 (1H, m, CHBr), 3.68-3.47 (3H, m, CH_2Br, CCl_3CH*H*), 3.24 (1H, dd, *J* = 15.8 and 5.4 Hz, CCl_3CH*H*), 2.72-2.49 (1H, m, CH*H*), 2.48-2.28 (1H, m, CH*H*); **^{13}C NMR** (50 MHz, $CDCl_3$) *δ*: 96.6, 62.3, 46.5, 41.5, 30.5; **MS** 330 $[M+H]^+$.

3-Bromo-1,1,1,4-tetrachlorobutane (16c)

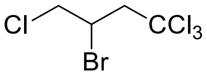

Yellow oil; 50% yield; **^{1}H NMR** (200 MHz, $CDCl_3$) *δ*: 4.52-4.40 (1H, m, CHBr), 4.08-3.99 (1H, m, CH*H*Cl), 3.88-3.77 (1H, m, CH*H*Cl), 3.60-3.49 (1H, m, CH*H*CCl_3), 3.34-3.22 (1H, m, CH*H*CCl_3); **^{13}C NMR** (50 MHz, $CDCl_3$) *δ*: 96.4, 58.9, 48.4, 44.8; **HRMS** exact mass calculated for $[M+H]^+$ ($C_4H_6BrCl_4^+$) requires *m/z* 272.8402, found *m/z* 272.8405.

4-Bromo-6,6,6-trichlorohexan-1-ol (16d)

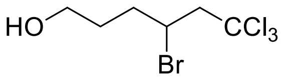

Yellow oil; 35% yield; **^{1}H NMR** (200 MHz, $CDCl_3$) δ: 4.38-4.27 (1H, m, CHBr), 3.65 (2H, t, J = 6.3 Hz, OCH_2), 3.44 (1H, dd, J = 15.8 and 5.1 Hz, $CCl_3CH\mathit{H}$), 3.20 (1H, dd, J = 15.8 and 5.3 Hz, $CCl_3CH\mathit{H}$), 2.60 (1H, br s, OH), 2.20-1.66 (4H, m, 2 x CH_2); **^{13}C NMR** (50 MHz, $CDCl_3$) δ: 96.9, 62.4, 61.5, 48.8, 35.8, 30.2; **HRMS** exact mass calculated for $C_6H_{10}BrCl_3NaO$ $[M+Na]^+$ 304.8873; found: 304.8864.

1-Bromo-2-(trichloromethyl)cyclooctane (16e)

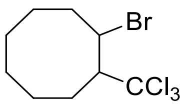

White solid; mp= 60-62 ^{o}C; 52% yield; Mixture of diastereoisomers (1:1); **^{1}H NMR** (200 MHz, $CDCl_3$) δ: 4.49-4.32 (1H, m, CHBr), 4.27-1.35 (13H, m, CH and 6 x CH_2); **^{13}C NMR** (50 MHz, $CDCl_3$) δ: 106.7, 106.7, 58.2, 58.0, 55.3, 54.9, 37.7, 36.4, 34.6, 32.9, 29.7, 29.5, 28.9, 28.3, 27.8, 27.5, 25.5, 23.1; **HRMS** exact mass calculated for $C_9H_{14}BrCl_3Na$ $[M+Na]^+$ 328.9231; found: 328.9225.

***N*-(3-Bromo-5,5,5-trichloropentyl)benzamide (16f)**

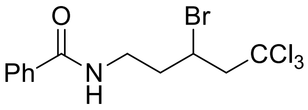

Yellow oil; 68% yield; **^{1}H NMR** (200 MHz, $CDCl_3$) δ: 7.75 (2H, d, J = 7.2 Hz, ArH), 7.53-7.28 (4H, m, ArH and NH), 4.39-4.27 (1H, m, CHBr), 3.75-3.49 (2H, m, NCH_2), 3.41 (1H, dd, J = 15.7 and 5.1 Hz, $CCl_3CH\mathit{H}$), 3.16 (1H, dd, J = 15.7 and 5.1 Hz, $CCl_3CH\mathit{H}$), 2.52-2.34 (1H, m, $CH\mathit{H}$), 2.18-2.02 (1H, m, $CH\mathit{H}$); **^{13}C NMR** (50 MHz,

$CDCl_3$) *δ*: 168.0, 134.1, 131.3, 128.3, 126.9, 96.7, 62.2, 46.0, 38.7, 38.2; **HRMS** exact mass calculated for $C_{12}H_{13}BrCl_3NO$ 369.9173; found: 369.9168.

4-Bromo-6,6,6-trichlorohexyl-benzoate (16g)

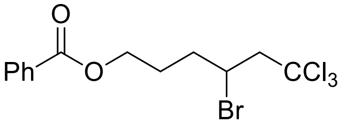

Yellow oil; 98% yield; **^{1}H NMR** (200 MHz, $CDCl_3$) *δ*: 8.02 (2H, d, *J* = 7.4 Hz, ArH), 7.58-7.37 (3H, m, ArH), 4.44-4.33 (3H, m, CHBr and 2 x OCH*H*), 3.45 (1H, dd, *J* = 15.8 and 4.9 Hz, CCl_3CH*H*), 3.22 (1H, dd, *J* = 15.8 and 5.6 Hz, CCl_3CH*H*), 2.25-1.96 (4H, m, 2 x CH_2); **^{13}C NMR** (50 MHz, $CDCl_3$) *δ*: 166.2, 132.9, 129.9, 129.4, 128.2, 96.8, 63.6, 62.3, 48.2, 35.8, 26.5; **HRMS** exact mass calculated for $C_{13}H_{14}BrCl_3NaO_2$ $[M+Na]^+$ 408.9135; found: 408.9122.

Determination of the Quantum Yield

Determination of the photon flux of the lamps

A 0.006M solution of potassium ferrioxalate was prepared by dissolving 120 mg of potassium ferrioxalate hydrate in 40 mL of 0.05M H_2SO_4. A buffered solution of phenanthroline was prepared by dissolving 10 mg of phenanthroline and 2.25 g of sodium acetate in 250 mL of 0.5 M H_2SO_4. Both solutions were stored in the dark. To determine the photon flux of the lamps, 2.0 mL of the solution of potassium ferrioxalate was placed in the cuvette, UV-Vis absorbance recorded (absorbance of interest at 510 nm) and irradiated for 90 seconds at the lamps. After irradiation, 0.35 mL of the phenanthroline solution was added to the cuvette. The solution was allowed to rest for 1 h (complete coordination of ferrous ions to phenanthroline). The absorbance of the solution was then measured at 510 nm.
The fraction of light absorbed (f) by this solution was calculated, using this absorbance (A):

$$f = 1 - 10^{-A} = 1 - 10^{-4.9987} = 0.9999$$

In order to measure the photon flux, the mol of Fe^{2+} are required:

$$\text{Mol } Fe^{2+} = \frac{V \times \Delta A}{l \times \varepsilon} = \frac{0.00235 \text{ L} \times 0.382}{1.0 \text{ cm} \times 11.100 \text{ L mol}^{-1} \text{ cm}^{-1}} = 8.09 \times 10^{-8} \text{ mol}$$

In this equation, V is the total volume of the solution after addition of the phenanthroline (0.00235 L), ΔA is the difference in the absorbance at 510 nm between the irradiated and the non-irradiated solutions, l is the path length (1.0 cm), and ε is the molar absorptivity at 510 nm (11.100 L mol^{-1} cm^{-1}). The photon flux was then calculated:

$$\text{Flux} = \frac{\text{Mol Fe}^{2+}}{\Phi \times t \times f} = \frac{8.09 \times 10^{-8}\ \text{mol}}{1.35 \times 90\ \text{sec} \times 0.9999} = 6.66 \times 10^{-10}\ \text{einsteins}^{-1}$$

In this equation, Φ is the quantum yield of the ferrioxalate actinometer,[19] t is the time of the irradiation (90 seconds), and f is the fraction of the light absorbed at the lamps (that is calculated above). Thus, the photon flux of the spectrophotometer was calculated to be 6.66×10^{-10} einstein s^{-1}.

Determination of the quantum yield of the bromoacetonitrile reaction

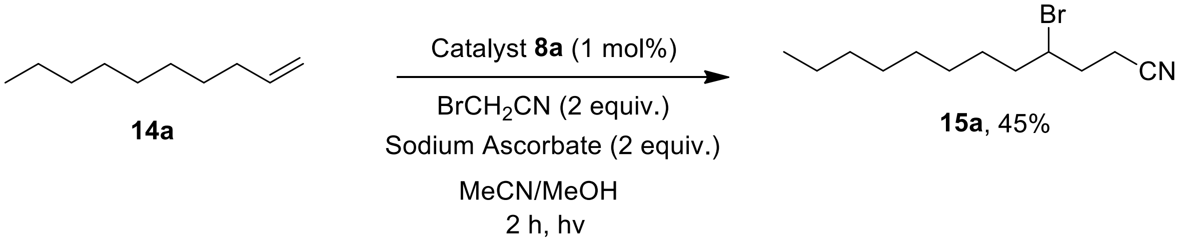

A cuvette was charged with 1-decene (14.0 mg, 0.10 mmol), $BrCH_2CN$ (24.0 mg, 0.20 mmol), sodium ascorbate (39.6 mg, 0.20 mmol) and **8a** (1.20 mg, 0.001 mmol) in methanol (0.6 mL) and acetonitrile (0.8 mL). The sample was stirred and then irradiated under CFL irradiation for 7200 s (2 h). After irradiation, the solvent was removed and the yield of the product was determined by ^1H NMR (45%). The quantum yield was determined with the following equation:

$$\Phi = \frac{\text{mol product}}{\text{flux} \times t \times f} = \frac{45 \times 10^{-6}\ \text{mol}}{6.66 \times 10^{-10}\ \text{einstein s}^{-1} \times 7200\ \text{s} \times 0.9999} = 9$$

Determination of the quantum yield of the bromotrichloromethane reaction

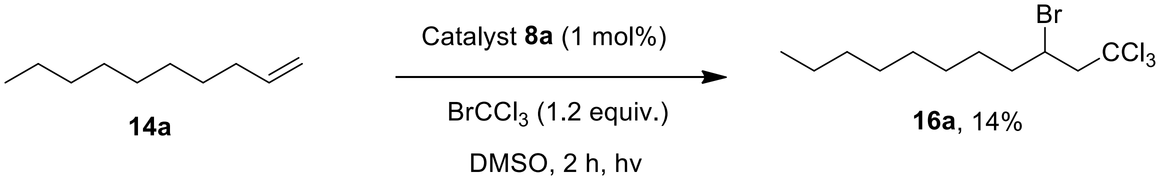

A cuvette was charged with 1-decene (14.0 mg, 0.10 mmol), $BrCCl_3$ (23.8 mg, 0.12 mmol) and **3c** (1.2 mg, 0.001 mmol) in DMSO (0.1 mL). The sample was stirred and then irradiated under CFL irradiation for 7200 s (2 h). After irradiation, the solvent was removed and the yield of the product was determined by ^{1}H NMR (14%). The quantum yield was determined with the following equation:

$$\Phi = \frac{\text{mol product}}{\text{flux} \times t \times f} = \frac{14 \times 10^{-6}\ \text{mol}}{6.66 \times 10^{-10}\ \text{einstein s}^{-1} \times 7200\ \text{s} \times 0.9999} = 3$$

Phosphorescence Quenching Studies

After irradiation of **8a** (10^{-3} M in DMF) at 370 nm, its phosphorescence was measured at 516 nm. Increasing the amount of the added 1-decene, no changes in the phosphorescence were observed.

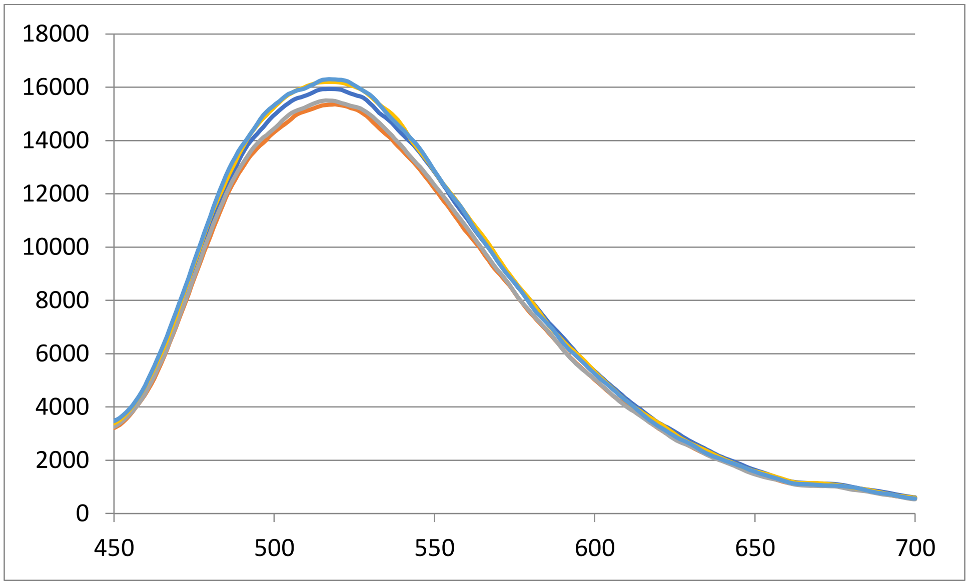

The corresponding Stern-Volmer plot is presented below**.**

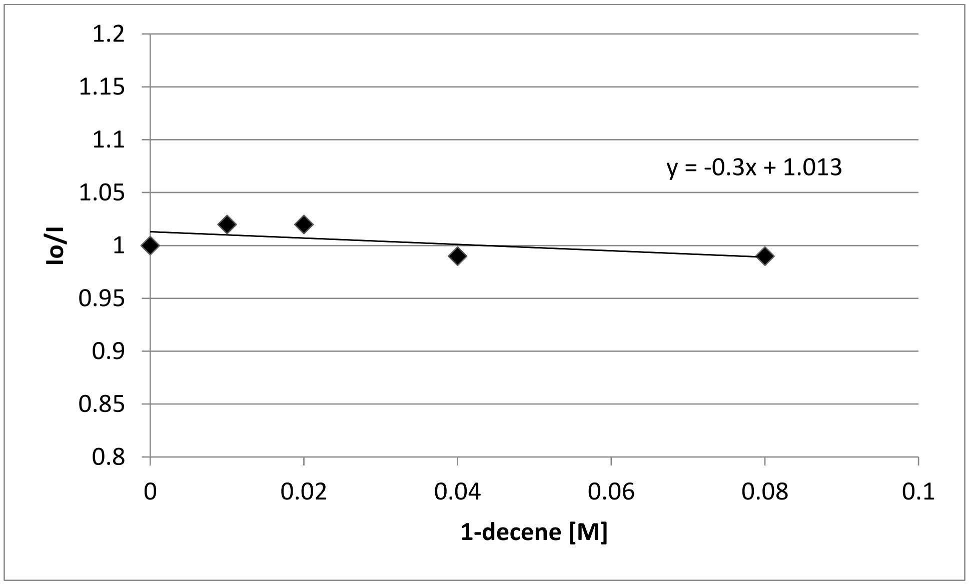

After irradiation of **8a** (10^{-3} M in DMF) at 370 nm, its phosphorescence was measured at 516 nm. Increasing the amount of the added $BrCH_2CN$, a constant decrease in the phosphorescence was observed.

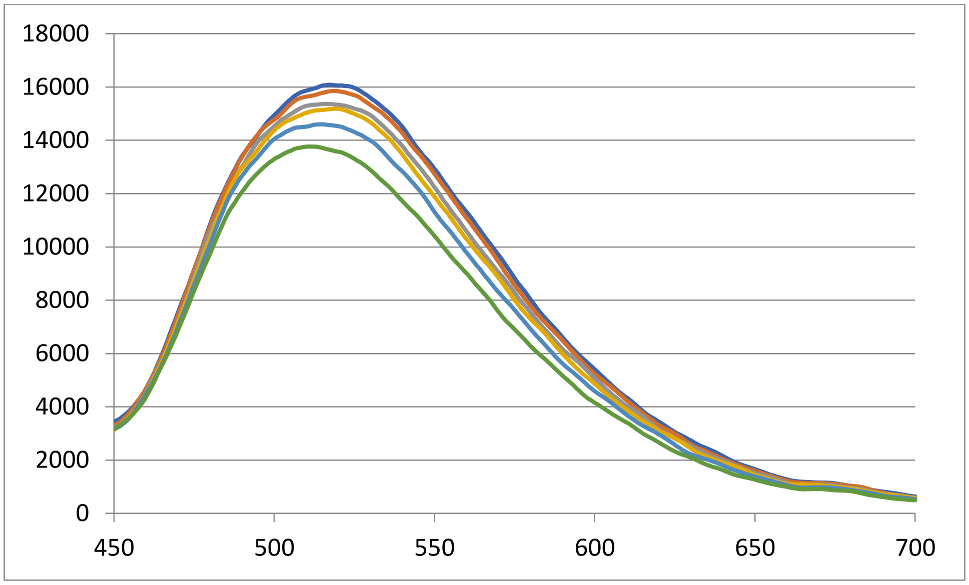

The corresponding Stern-Volmer plot is presented below**.**

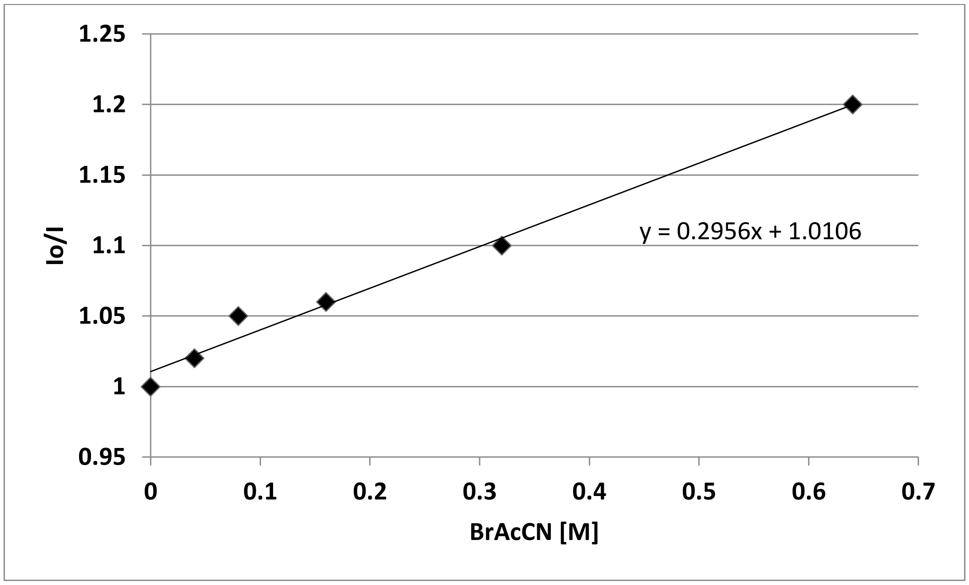

After irradiation of **8a** (10^{-3} M in DMF) at 370 nm, its phosphorescence was measured at 516 nm. Increasing the amount of the added sodium ascorbate, a constant decrease in the phosphorescence was observed.

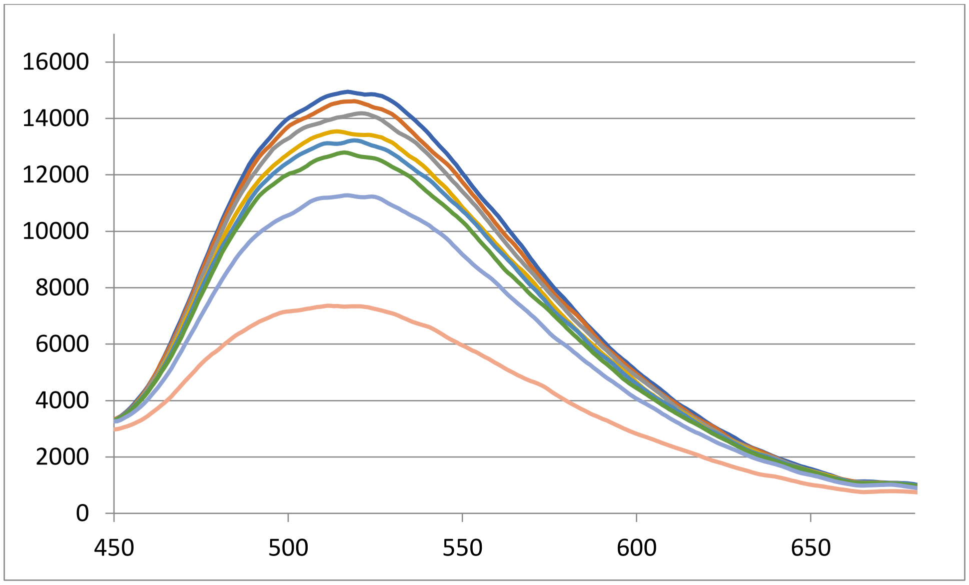

The corresponding Stern-Volmer plot is presented below**.**

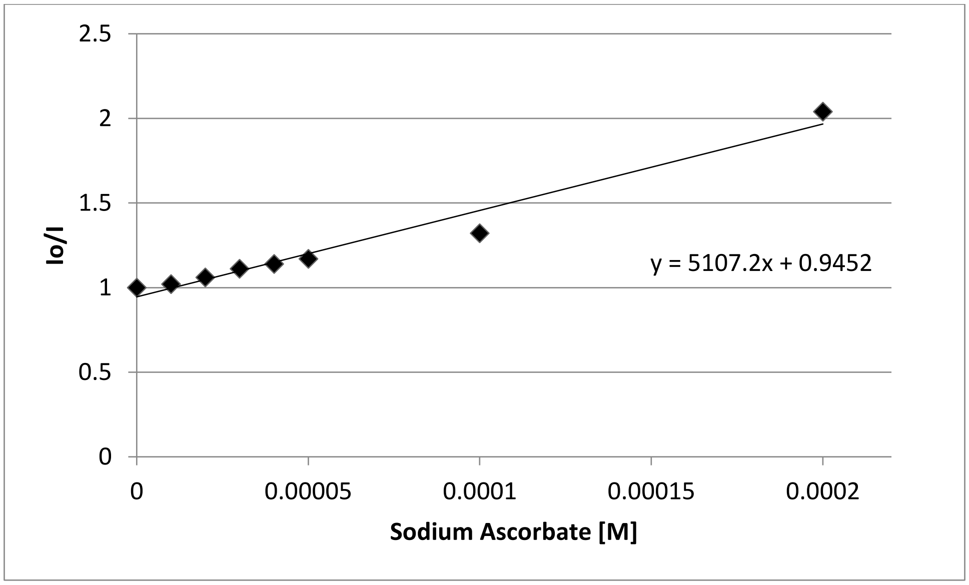

After irradiation of **8a** (10^{-3} M in DMSO) at 370 nm, its phosphorescence was measured at 516 nm. Increasing the amount of the added 1-decene, no changes in the phosphorescence was observed.

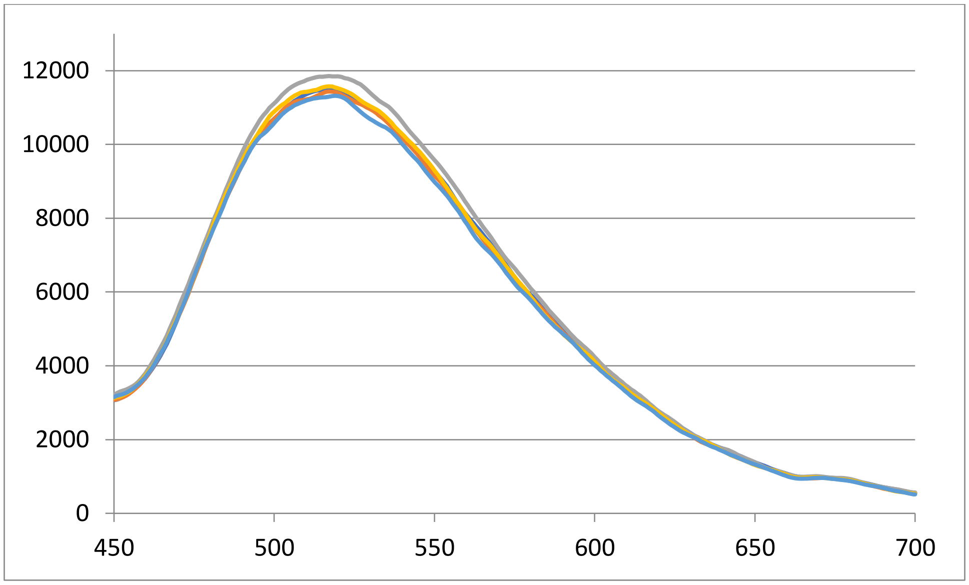

The corresponding Stern-Volmer plot is presented below**.**

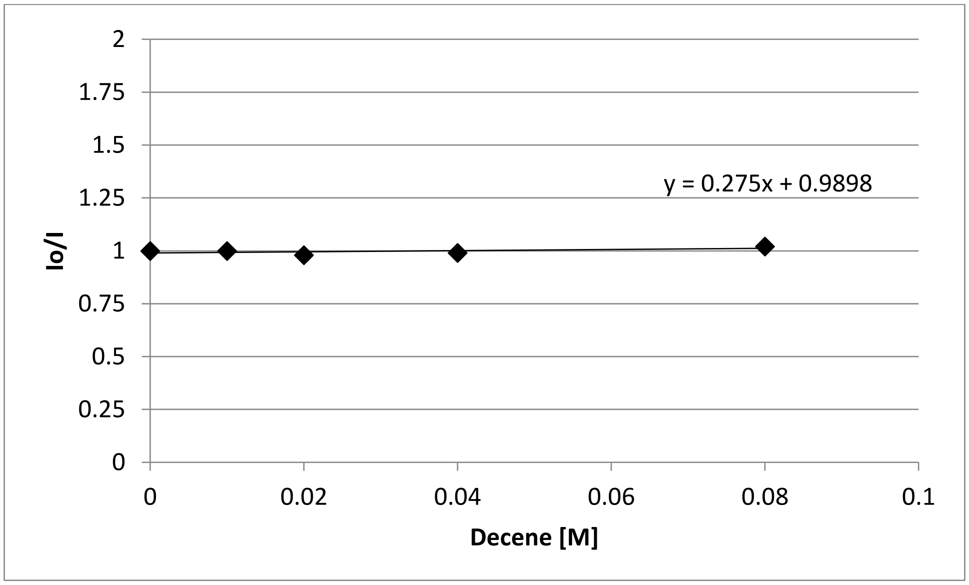

After irradiation of **8a** (10^{-3} M in DMSO) at 370 nm, its phosphorescence was measured at 516 nm. Increasing the amount of the added $BrCCl_3$, a constant decrease in the phosphorescence was observed.

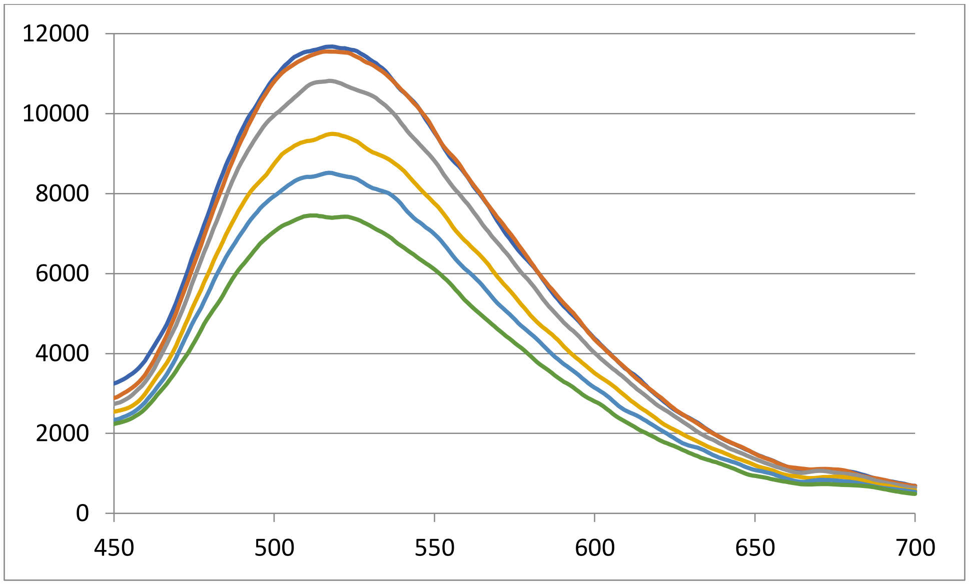

The corresponding Stern-Volmer plot is presented below**.**

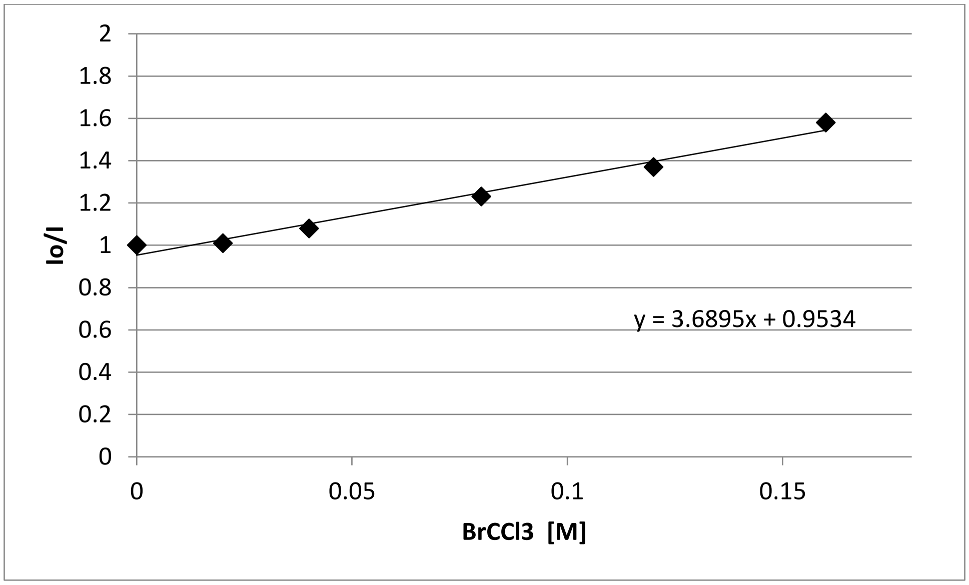

References

1. G. Sprintschnik, H. W. Sprintschnik, P. P. Kirsch, D. G. Whitten, *J. Am. Chem. Soc.*, **1977**, *99*, 4947.
2. G. V. Vougioukalakis, T. Stergiopoulos, A. G. Kontos, E. K. Pefkianakis, K. Papadopoulos, P. Falaras, *Dalton Trans.*, **2013**, *42*, 6582.
3. E. K. Pefkianakis, D. Christodouleas, D. L. Giokas, K. Papadopoulos, G. C. Vougioukalakis, *Eur. J. Inorg. Chem.*, **2013**, 4628.
4. A. Stublla, P. G. Potvin, *Eur. J. Inorg. Chem.*, **2010**, 3040.
5. K. Heinze, K. Hempel, A. Breivogel, *Z. Anorg. Allg. Chem*, **2009**, *635*, 2541.
6. Y. Zhou, H.-Y. Zhang, Z. Y. Zhang, Y. Liu, *J. Am. Chem. Soc.*, **2017**, *139*, 7168.
7. A. M. Johns, T. S. Ahmed, B. W. Jackson, R. H. Grubbs, R. L. Pederson, *Org. Lett.*, **2016**, *18*, 772.
8. I. G. Molnar, R. Gilmour, *J. Am. Chem. Soc.*, **2016**, *138*, 5004.
9. J. Breienfeld, J. Ruiz, M. D. Wodrich, X. Hu, *J. Am. Chem. Soc.*, **2013**, *13*5, 12004.
10. X. Wang, Y. Ye, S. Zhang, J. Feng, Y. Xu, Y. Zhang, J. Wang, *J. Am. Chem. Soc.*, **2011**, *13*3, 16410.
11. I. Triandafillidi, M. G. Kokotou, C. G. Kokotos, *Org. Lett.*, **2018**, *20,* 36
12. R. Gawin, A. Kozakiewicz, P. A. Gunka, P. Dabrowski, K. Skowerski, *Angew. Chem. Int. Ed.*, **2016**, *56*, 981.
13. S. Mizuta, S. Verhoog, K. M. Eagle, T. Khotavivattana, M. O'Duill, K. Wheelhouse, G. Rassias, M. Medebielle, V. Gouverneur, *J. Am. Chem. Soc.*, **2013**, *135*, 2505
14. E. Voutyritsa, I. Triandafillidi, C. G. Kokotos, *ChemCatChem*, **2018**, *10*, 2466.
15. D. Meyer, E. Vin, B. Wyler, G. Lapointe, P. Rehaud, *Synlett*, **2016**, *27*, 745.
16. T. Yajima, M. Ikegami, *Eur. J. Org. Chem.*, **2017**, 2017, 2126.
17. J. Tsuji, K. Sato, H. Nagashima, *Tetrahedron,* **1985**, *41*, 393.
18. L. Mathew, J. Warkentin, *Can. J. Chem.*, **1988**, *66*, 11.

19. (a) J. Lee, H. H. Seliger, *J. Chem. Phys.*, **1964**, *40*, 519. (b) J. N. Demas, W. D. Bowman, E. F. Zalewskl, R. A. Velapoldl, *J. Phys. Chem*. **1981**, *85*, 2766. (c) J. R. Bolton, M. I. Stefan, P.-S. Shaw, K. R. Lykke, *J. Photochem. Photobiol. A: Chemistry*, **2011**, *222*, 166.

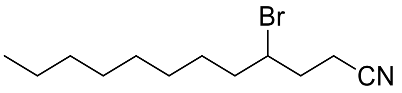
Br
CN

15a

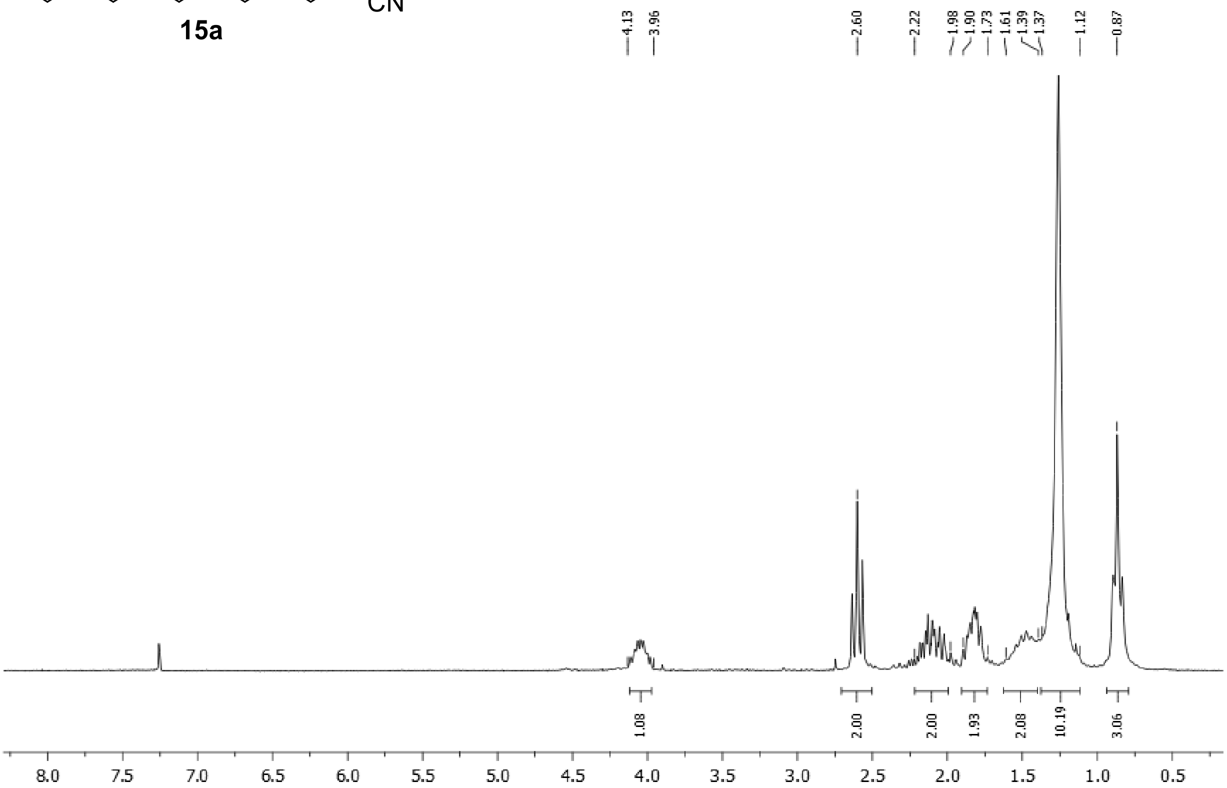
4.13
3.96
2.60
2.22
1.98
1.90
1.73
1.61
1.39
1.37
1.12
0.87
1.08
2.00
2.00
1.93
2.08
10.19
3.06
8.0 7.5 7.0 6.5 6.0 5.5 5.0 4.5 4.0 3.5 3.0 2.5 2.0 1.5 1.0 0.5

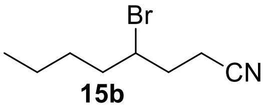
Br
CN

15b

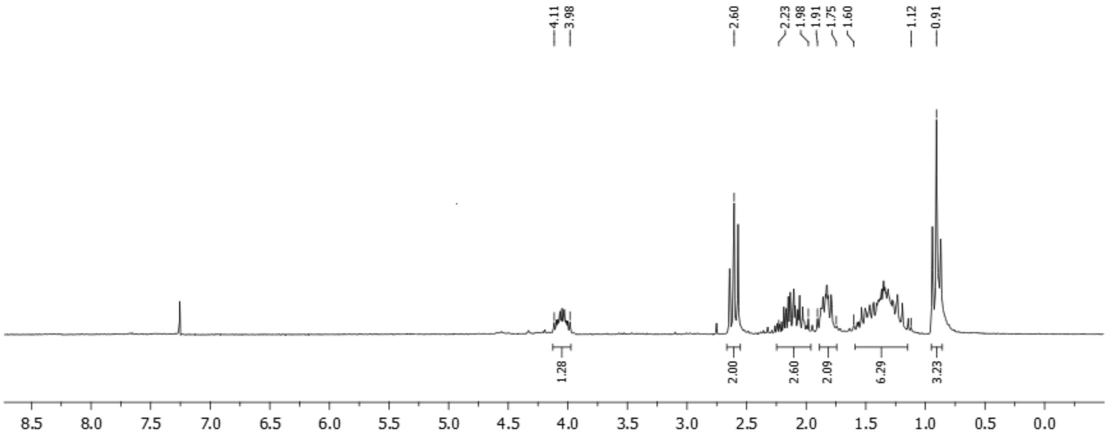
4.11
3.98
2.60
2.23
1.98
1.91
1.75
1.60
1.12
0.91
1.28
2.00
2.60
2.09
6.29
3.23
8.5 8.0 7.5 7.0 6.5 6.0 5.5 5.0 4.5 4.0 3.5 3.0 2.5 2.0 1.5 1.0 0.5 0.0

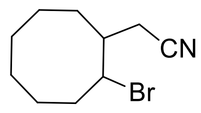
CN
Br

15c

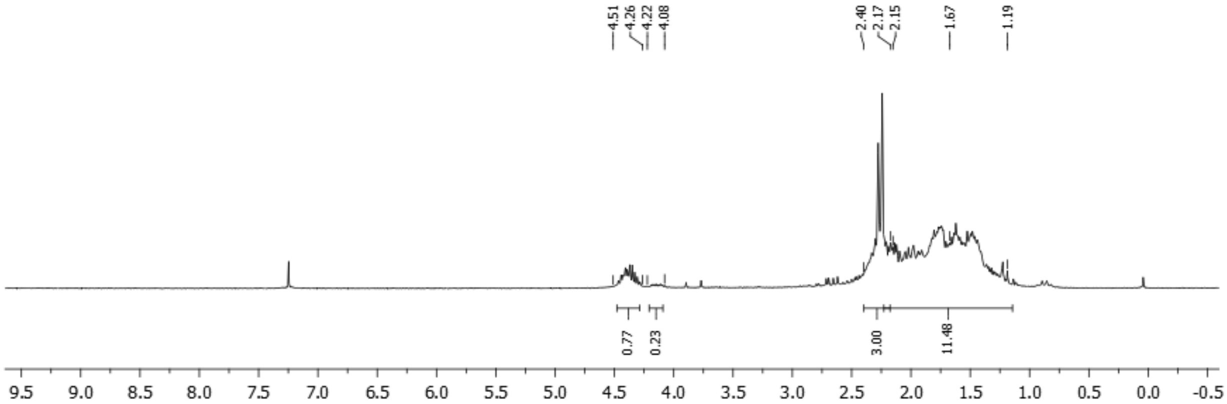
4.51
4.26
4.22
4.08
2.40
2.17
2.15
1.67
1.19
0.77
0.23
3.00
11.48
9.5 9.0 8.5 8.0 7.5 7.0 6.5 6.0 5.5 5.0 4.5 4.0 3.5 3.0 2.5 2.0 1.5 1.0 0.5 0.0 -0.5

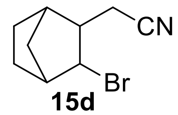
CN
Br

15d

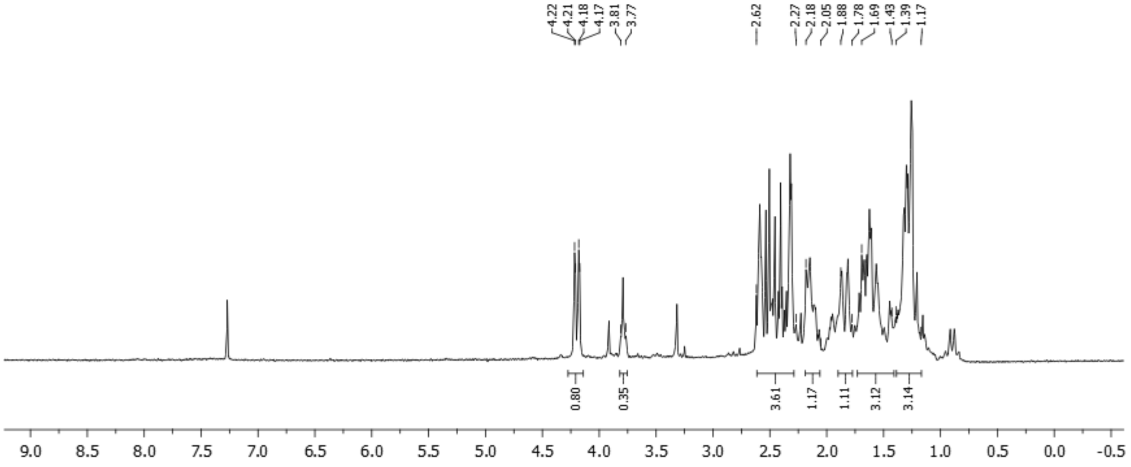
4.22
4.21
4.18
4.17
3.81
3.77
2.62
2.27
2.18
2.05
1.88
1.78
1.69
1.43
1.39
1.17
0.80
0.35
3.61
1.17
1.11
3.12
3.14
9.0 8.5 8.0 7.5 7.0 6.5 6.0 5.5 5.0 4.5 4.0 3.5 3.0 2.5 2.0 1.5 1.0 0.5 0.0 -0.5

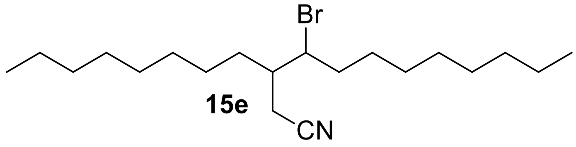
Br
CN

15e

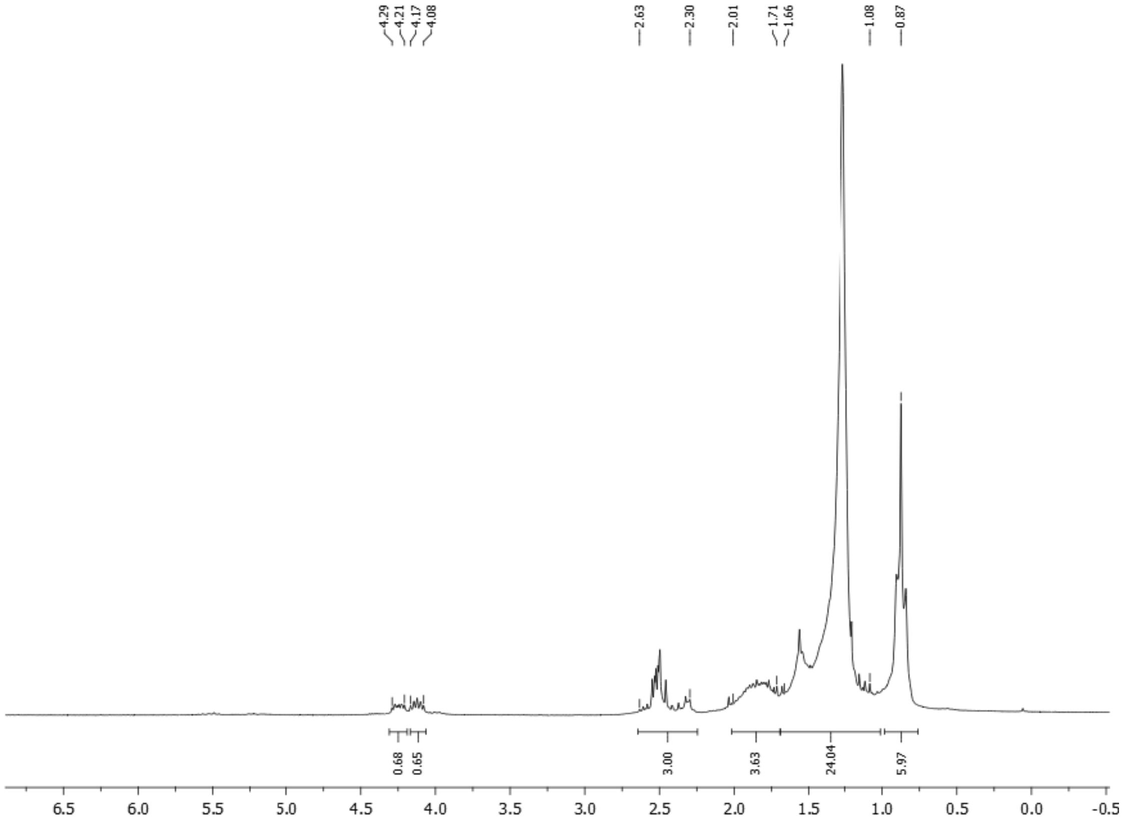
4.29
4.21
4.17
4.08
2.63
2.30
2.01
1.71
1.66
1.08
0.87
0.68
0.65
3.00
3.63
24.04
5.97
6.5
6.0
5.5
5.0
4.5
4.0
3.5
3.0
2.5
2.0
1.5
1.0
0.5
0.0
-0.5

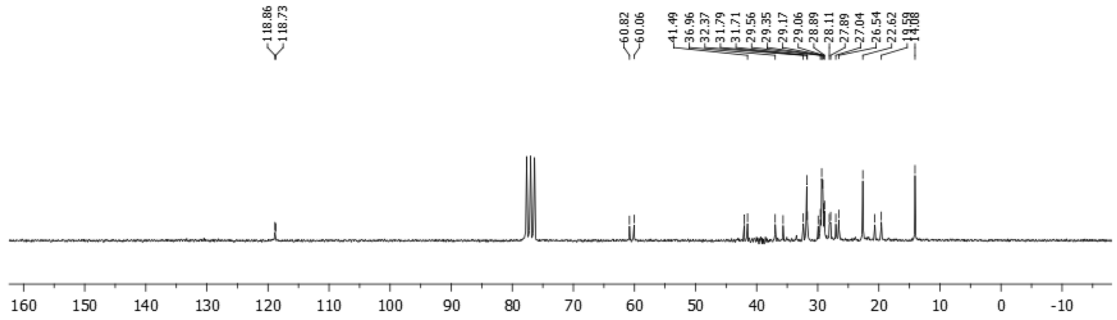
118.86
118.73
60.82
60.06
41.49
36.96
32.37
31.79
31.71
29.56
29.35
29.17
29.06
28.89
28.11
27.89
27.04
26.54
22.62
14.08
160
150
140
130
120
110
100
90
80
70
60
50
40
30
20
10
0
-10

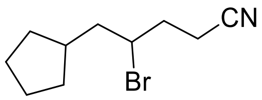
CN
Br

15f

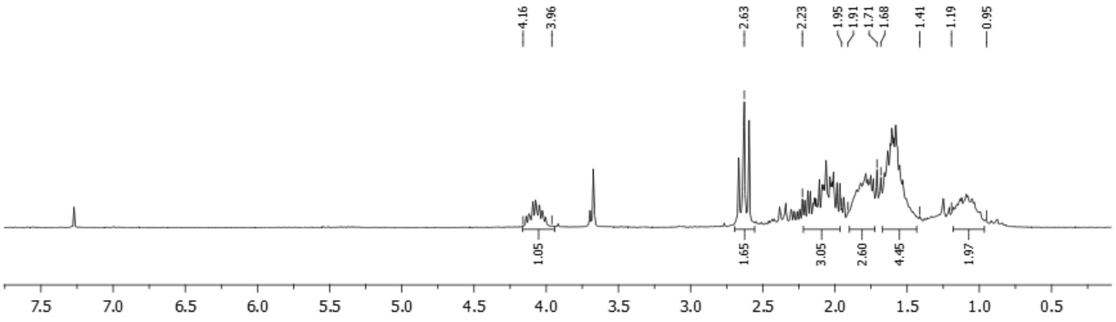
4.16
3.96
2.63
2.23
1.95
1.91
1.71
1.68
1.41
1.19
0.95
1.05
1.65
3.05
2.60
4.45
1.97
7.5 7.0 6.5 6.0 5.5 5.0 4.5 4.0 3.5 3.0 2.5 2.0 1.5 1.0 0.5

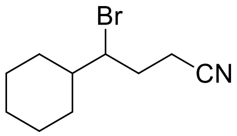
Br
CN

15g

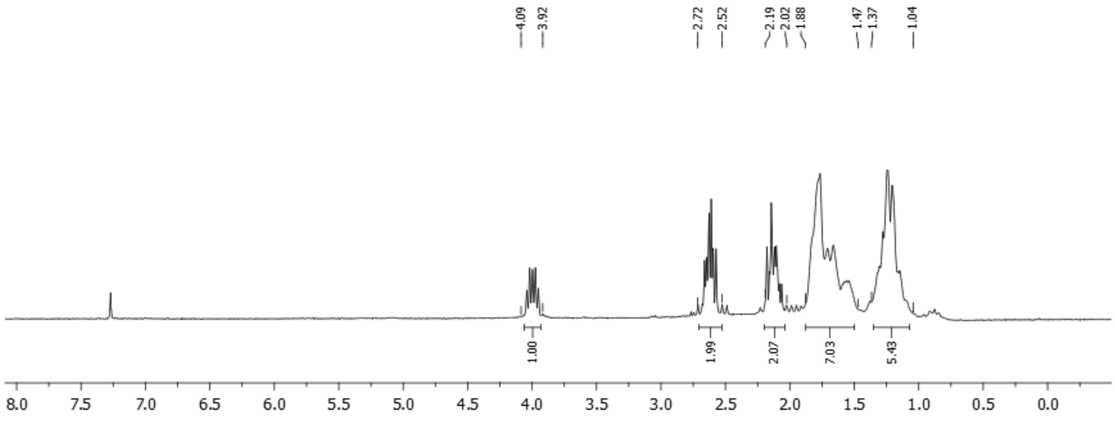
4.09
3.92
2.72
2.52
2.19
2.02
1.88
1.47
1.37
1.04
1.00
1.99
2.07
7.03
5.43
8.0 7.5 7.0 6.5 6.0 5.5 5.0 4.5 4.0 3.5 3.0 2.5 2.0 1.5 1.0 0.5 0.0

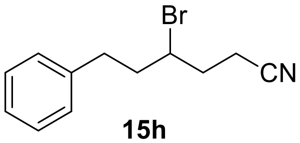
Br
CN

15h

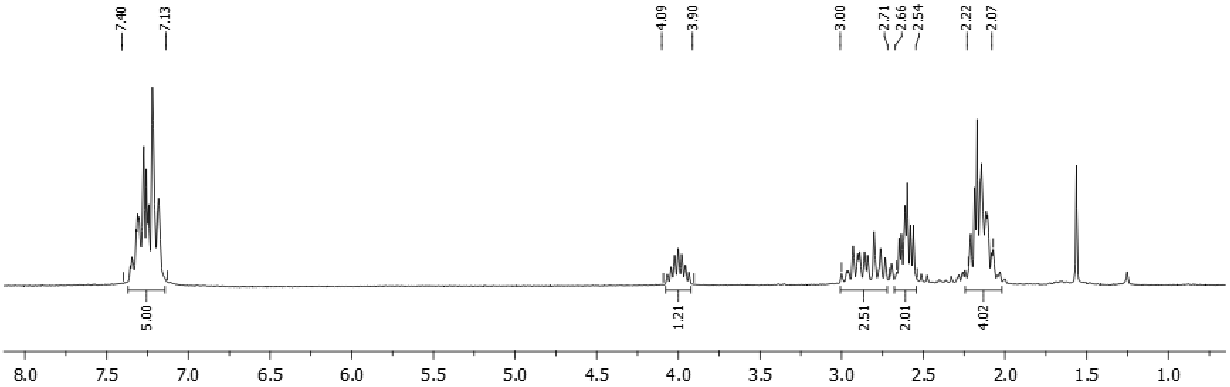
7.40
7.13
4.09
3.90
3.00
2.71
2.66
2.54
2.22
2.07
5.00
1.21
2.51
2.01
4.02
8.0
7.5
7.0
6.5
6.0
5.5
5.0
4.5
4.0
3.5
3.0
2.5
2.0
1.5
1.0

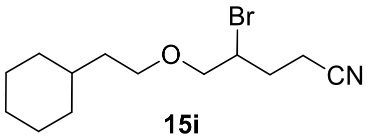
Br
O
CN

15i

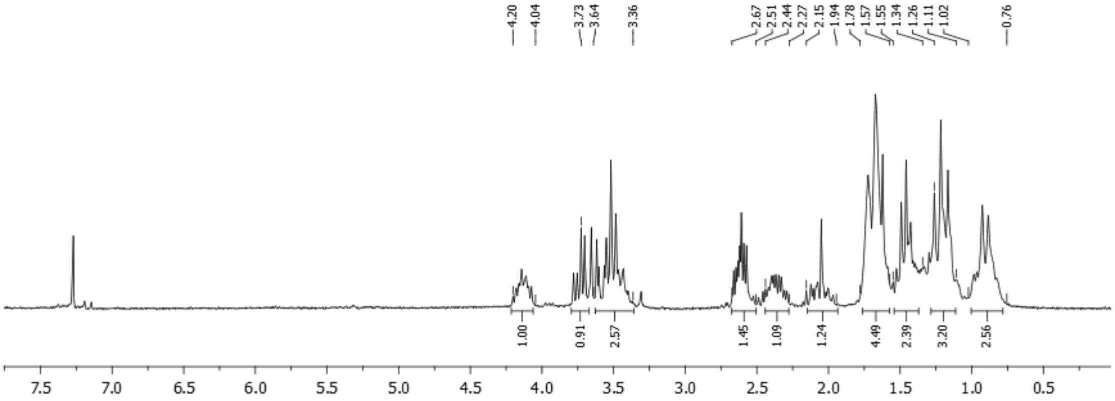
4.20
4.04
3.73
3.64
3.36
2.67
2.51
2.44
2.27
2.15
1.94
1.78
1.57
1.55
1.34
1.26
1.11
1.02
0.76
1.00
0.91
2.57
1.45
1.09
1.24
4.49
2.39
3.20
2.56
7.5
7.0
6.5
6.0
5.5
5.0
4.5
4.0
3.5
3.0
2.5
2.0
1.5
1.0
0.5

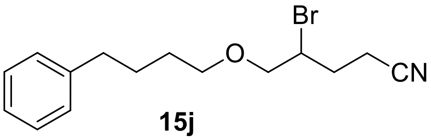
Br
O
CN

15j

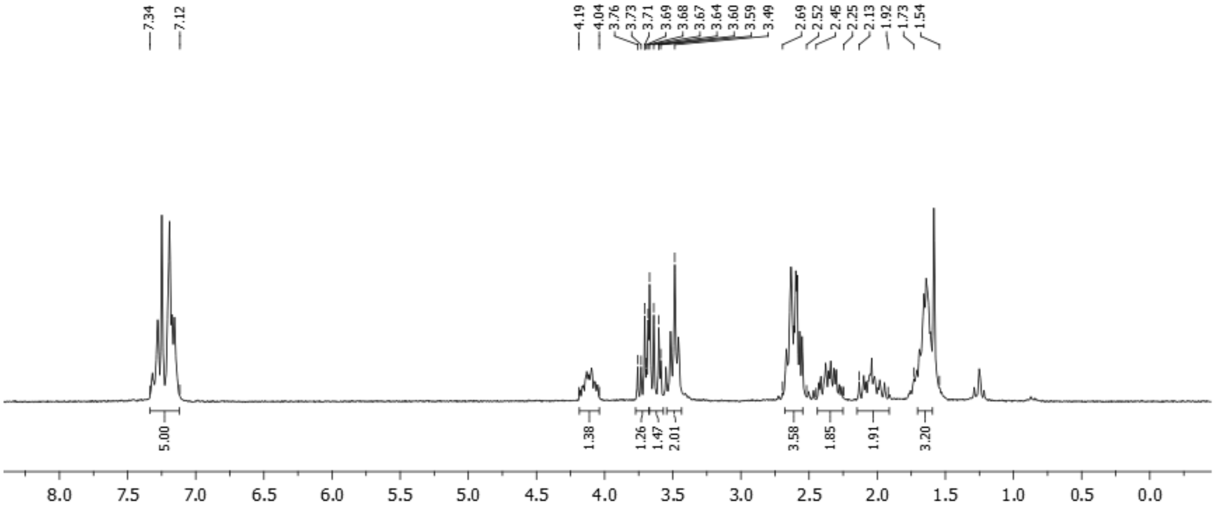
7.34
7.12
4.19
4.04
3.76
3.73
3.71
3.69
3.68
3.67
3.64
3.60
3.59
3.49
2.69
2.52
2.45
2.25
2.13
1.92
1.73
1.54
5.00
1.38
1.26
1.47
2.01
3.58
1.85
1.91
3.20
8.0
7.5
7.0
6.5
6.0
5.5
5.0
4.5
4.0
3.5
3.0
2.5
2.0
1.5
1.0
0.5
0.0

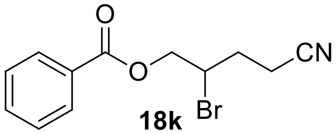
O
O
CN
Br

18k

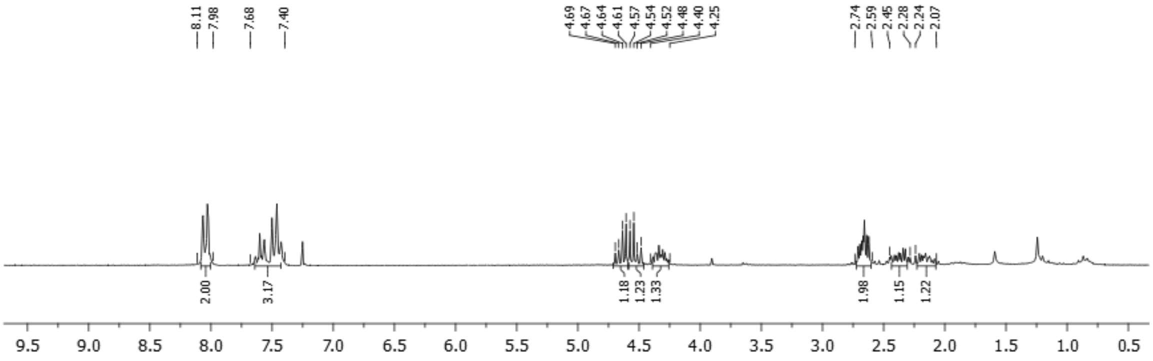
8.11
7.98
7.68
7.40
4.69
4.67
4.64
4.61
4.57
4.54
4.52
4.48
4.40
4.25
2.74
2.59
2.45
2.28
2.24
2.07
2.00
3.17
1.18
1.23
1.33
1.98
1.15
1.22
9.5
9.0
8.5
8.0
7.5
7.0
6.5
6.0
5.5
5.0
4.5
4.0
3.5
3.0
2.5
2.0
1.5
1.0
0.5

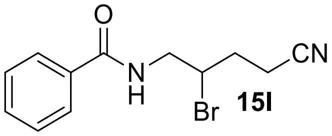
O
N
H
CN
Br

15l

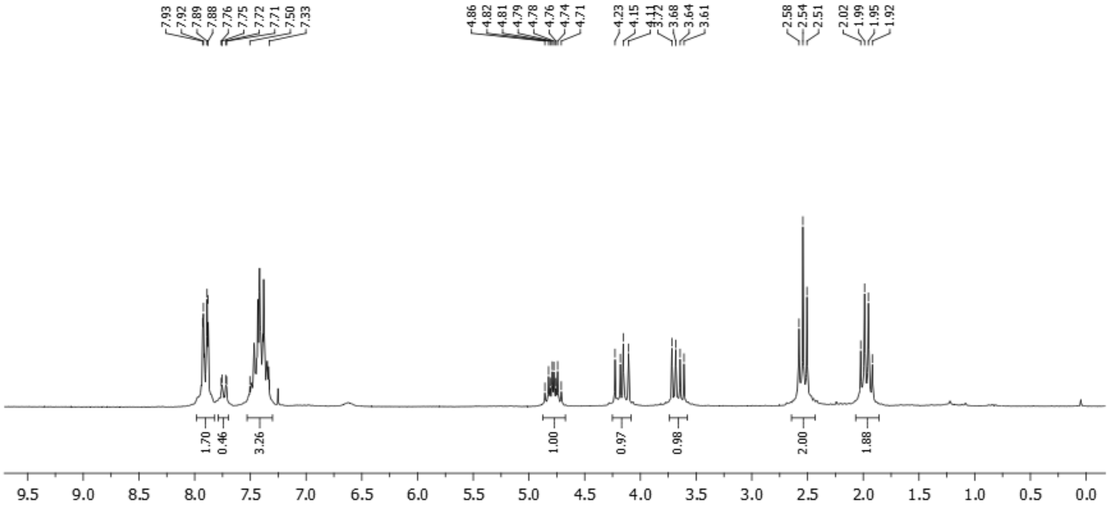
7.93
7.92
7.89
7.88
7.76
7.75
7.72
7.71
7.50
7.33
4.86
4.82
4.81
4.79
4.78
4.76
4.74
4.71
4.23
4.15
4.11
3.72
3.68
3.64
3.61
2.58
2.54
2.51
2.02
1.99
1.95
1.92
1.70
0.46
3.26
1.00
0.97
0.98
2.00
1.88
9.5 9.0 8.5 8.0 7.5 7.0 6.5 6.0 5.5 5.0 4.5 4.0 3.5 3.0 2.5 2.0 1.5 1.0 0.5 0.0

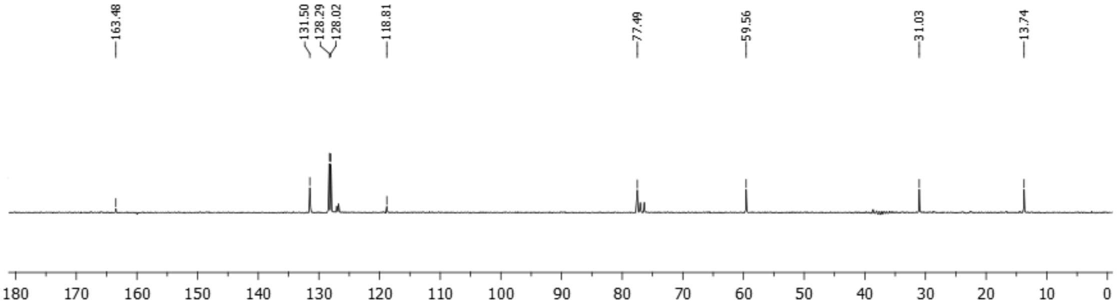
163.48
131.50
128.29
128.02
118.81
77.49
59.56
31.03
13.74
180 170 160 150 140 130 120 110 100 90 80 70 60 50 40 30 20 10 0

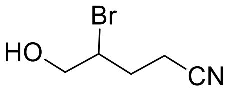
Br
HO
CN

15m

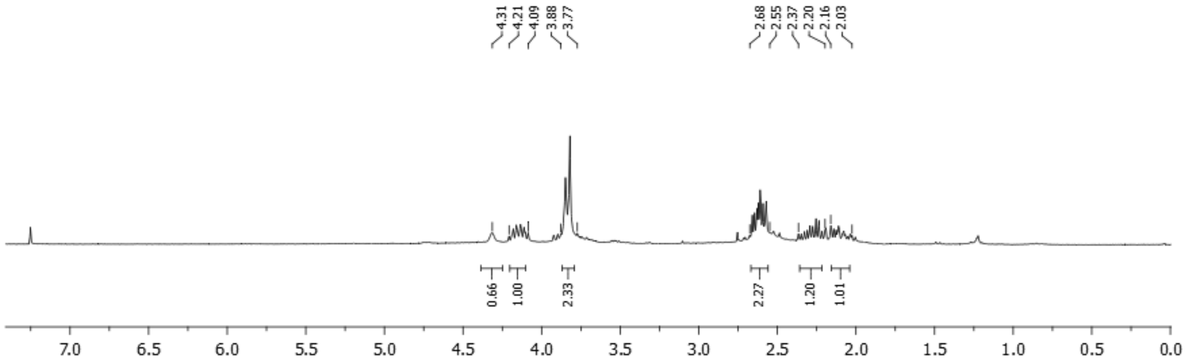
4.31
4.21
4.09
3.88
3.77
2.68
2.55
2.37
2.20
2.16
2.03
0.66
1.00
2.33
2.27
1.20
1.01
7.0 6.5 6.0 5.5 5.0 4.5 4.0 3.5 3.0 2.5 2.0 1.5 1.0 0.5 0.0

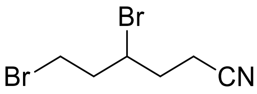
Br
Br
CN

15n

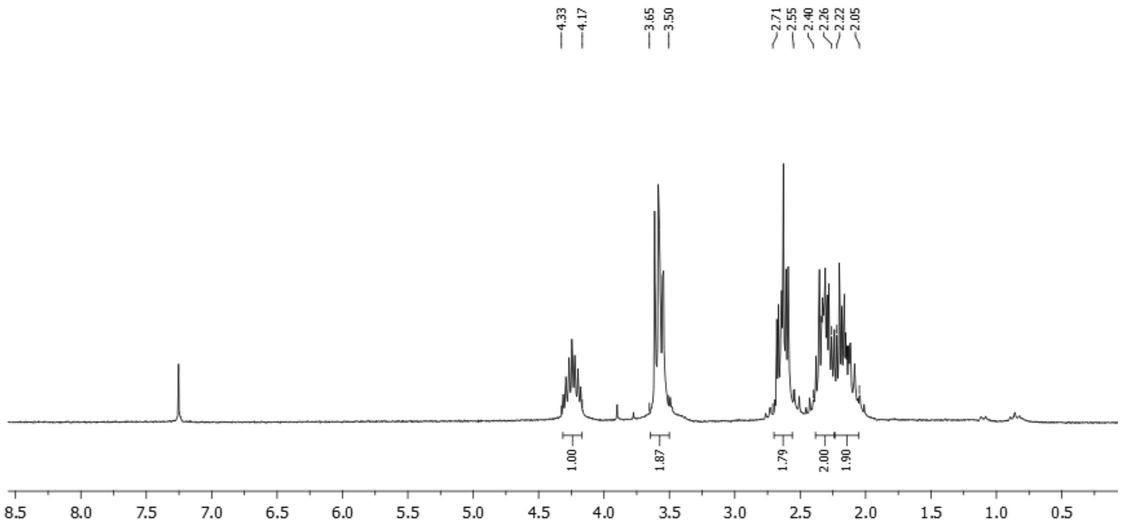
4.33
4.17
3.65
3.50
2.71
2.55
2.40
2.26
2.22
2.05
1.00
1.87
1.79
2.00
1.90
8.5 8.0 7.5 7.0 6.5 6.0 5.5 5.0 4.5 4.0 3.5 3.0 2.5 2.0 1.5 1.0 0.5

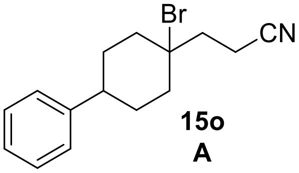
Br
CN

15o
A

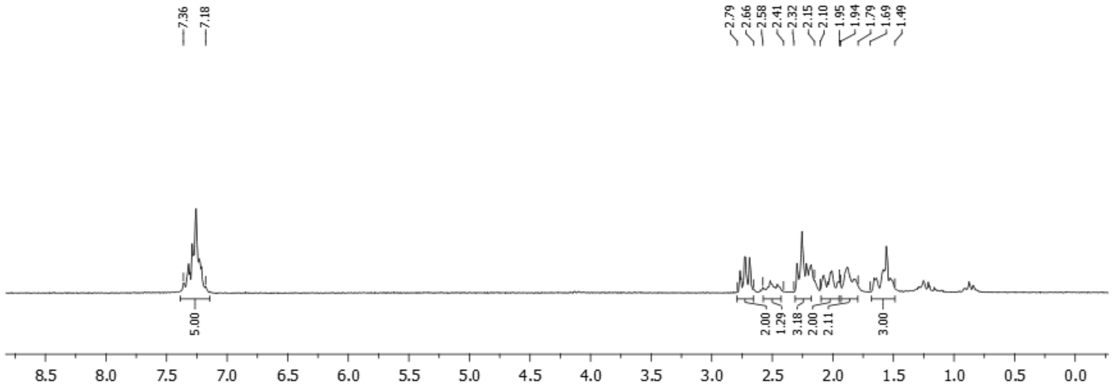
7.36
7.18
2.79
2.66
2.58
2.41
2.32
2.15
2.10
1.95
1.94
1.79
1.69
1.49
5.00
2.00
1.29
3.18
2.00
2.11
3.00
8.5 8.0 7.5 7.0 6.5 6.0 5.5 5.0 4.5 4.0 3.5 3.0 2.5 2.0 1.5 1.0 0.5 0.0

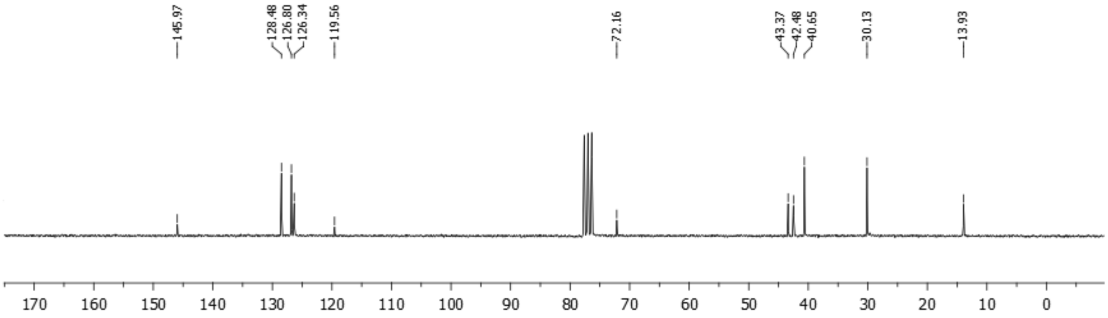
145.97
128.48
126.80
126.34
119.56
72.16
43.37
42.48
40.65
30.13
13.93
170 160 150 140 130 120 110 100 90 80 70 60 50 40 30 20 10 0

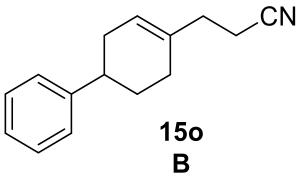
CN

15o
B

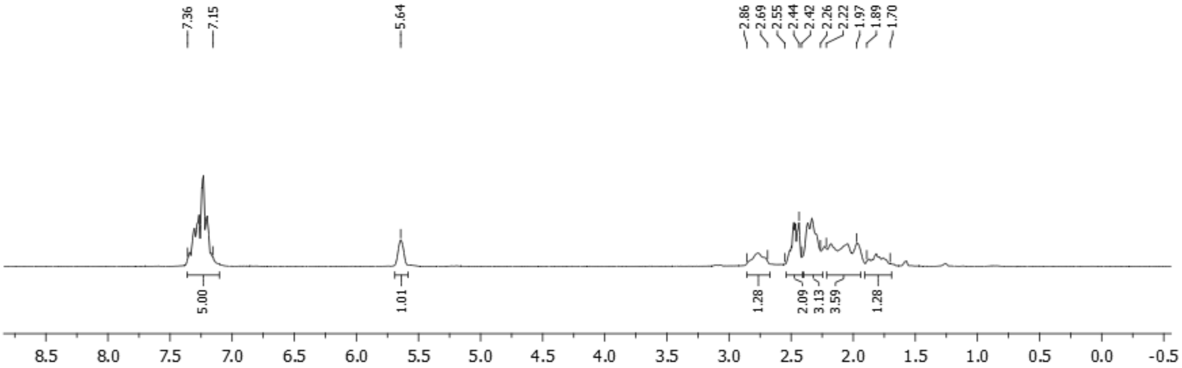
7.36
7.15
5.64
2.86
2.69
2.55
2.44
2.42
2.26
2.22
1.97
1.89
1.70
5.00
1.01
1.28
2.09
3.13
3.59
1.28
8.5 8.0 7.5 7.0 6.5 6.0 5.5 5.0 4.5 4.0 3.5 3.0 2.5 2.0 1.5 1.0 0.5 0.0 -0.5

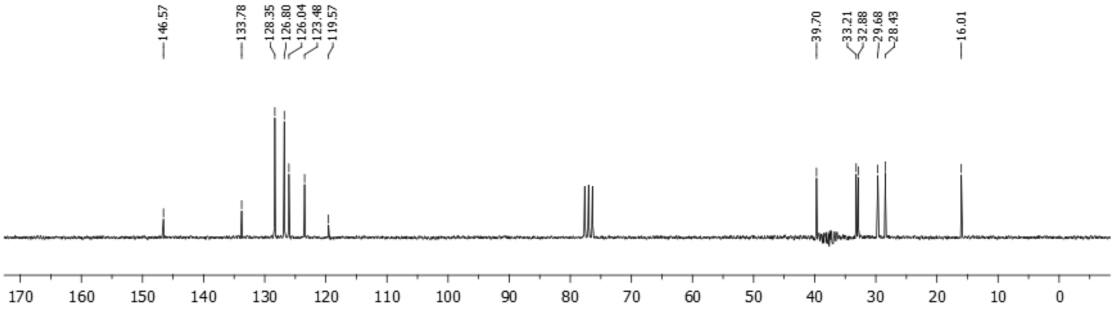
146.57
133.78
128.35
126.80
126.04
123.48
119.57
39.70
33.21
32.88
29.68
28.43
16.01
170 160 150 140 130 120 110 100 90 80 70 60 50 40 30 20 10 0

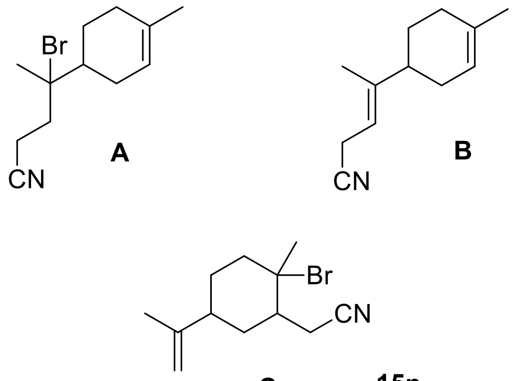
Br
CN
A
CN
B
Br
CN
C

15p

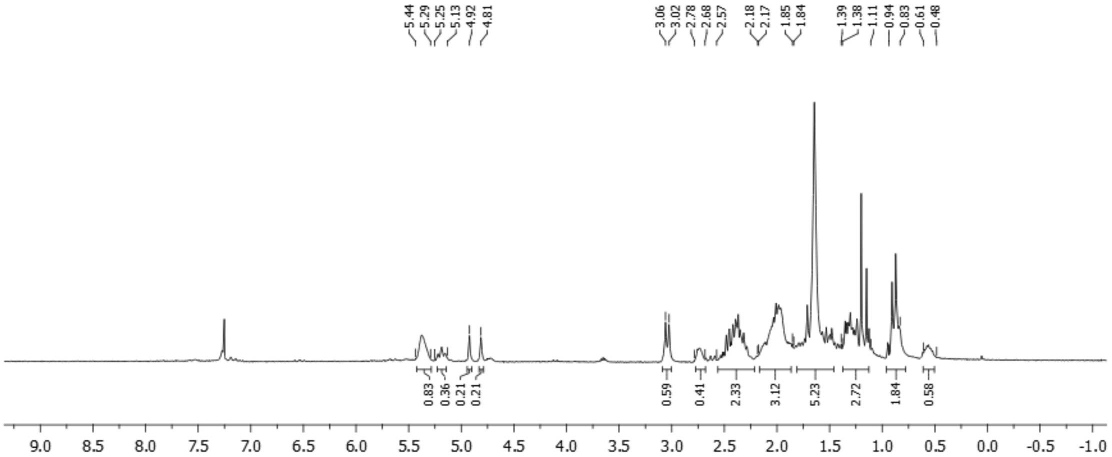
5.44
5.29
5.25
5.13
4.92
4.81
3.06
3.02
2.78
2.68
2.57
2.18
2.17
1.85
1.84
1.39
1.38
1.11
0.94
0.83
0.61
0.48
0.83
0.36
0.21
0.21
0.59
0.41
2.33
3.12
5.23
2.72
1.84
0.58
9.0 8.5 8.0 7.5 7.0 6.5 6.0 5.5 5.0 4.5 4.0 3.5 3.0 2.5 2.0 1.5 1.0 0.5 0.0 -0.5 -1.0

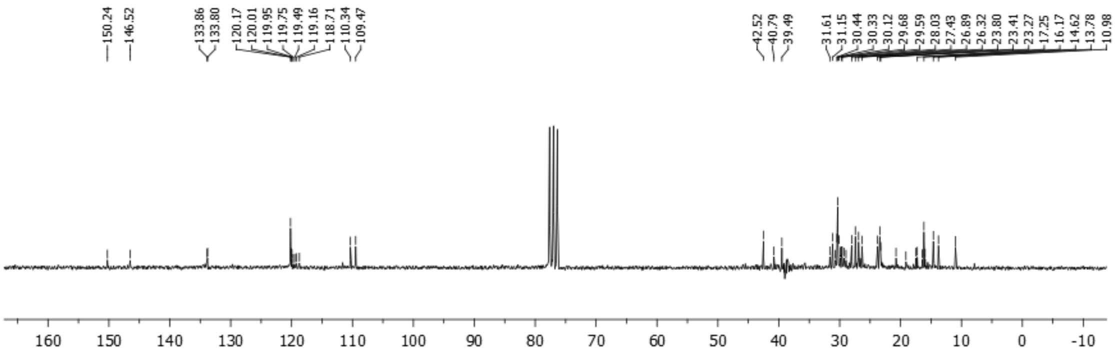
150.24
146.52
133.86
133.80
120.17
120.01
119.95
119.75
119.49
119.16
118.71
110.34
109.47
42.52
40.79
39.49
31.61
31.15
30.44
30.33
30.12
29.68
29.59
28.03
27.43
26.89
26.32
23.80
23.41
23.27
17.25
16.17
14.62
13.78
10.98
160 150 140 130 120 110 100 90 80 70 60 50 40 30 20 10 0 -10

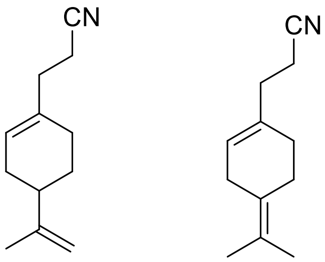
CN
CN

A 15q B

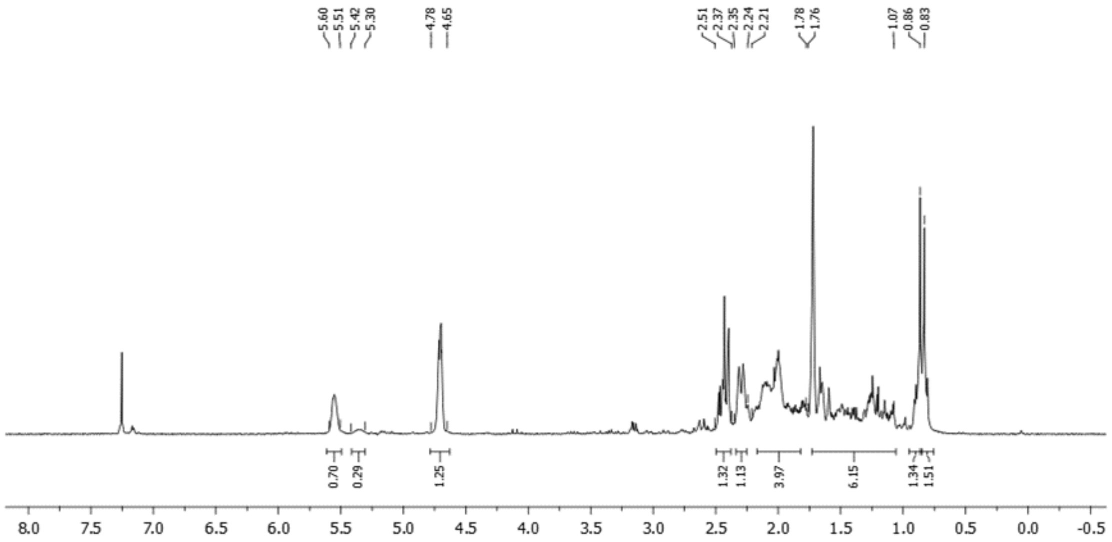
5.60
5.51
5.42
5.30
4.78
4.65
2.51
2.37
2.35
2.24
2.21
1.78
1.76
1.07
0.86
0.83
0.70
0.29
1.25
1.32
1.13
3.97
6.15
1.34
1.51
8.0 7.5 7.0 6.5 6.0 5.5 5.0 4.5 4.0 3.5 3.0 2.5 2.0 1.5 1.0 0.5 0.0 -0.5

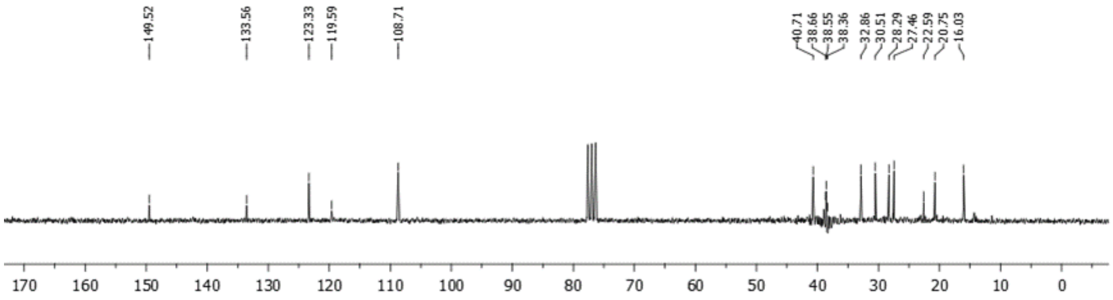
149.52
133.56
123.33
119.59
108.71
40.71
38.66
38.55
38.36
32.86
30.51
28.29
27.46
22.59
20.75
16.03
170 160 150 140 130 120 110 100 90 80 70 60 50 40 30 20 10 0

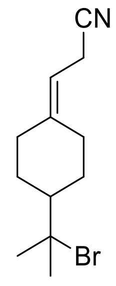
CN
Br

15q

C

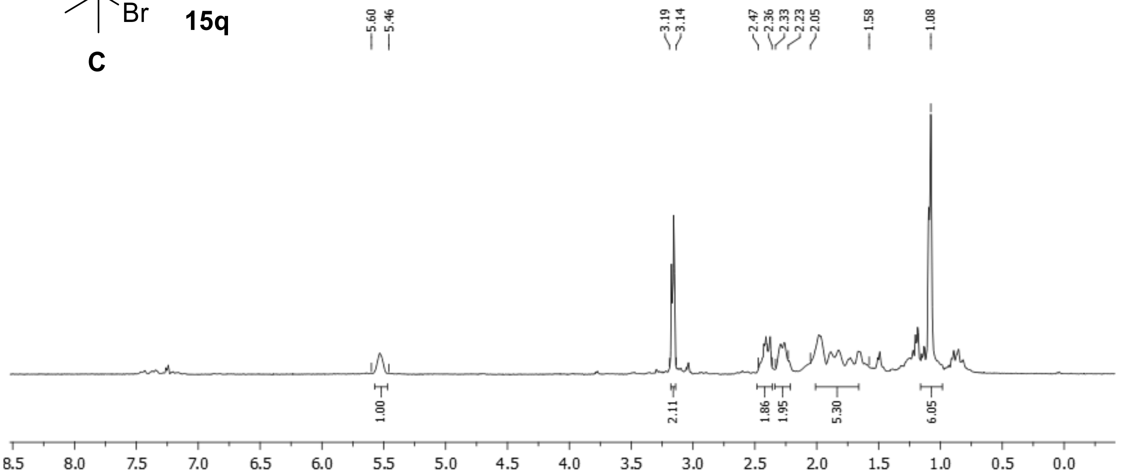
5.60
5.46
3.19
3.14
2.47
2.36
2.33
2.23
2.05
1.58
1.08
1.00
2.11
1.86
1.95
5.30
6.05
8.5 8.0 7.5 7.0 6.5 6.0 5.5 5.0 4.5 4.0 3.5 3.0 2.5 2.0 1.5 1.0 0.5 0.0

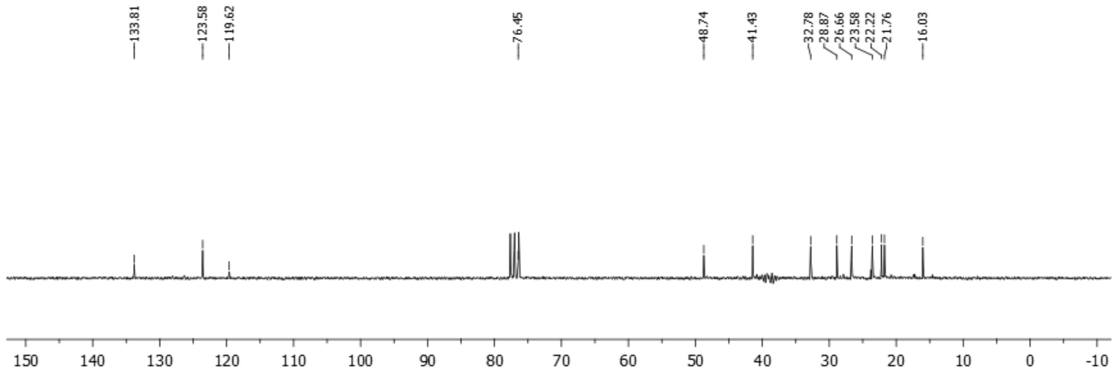
133.81
123.58
119.62
76.45
48.74
41.43
32.78
28.87
26.66
23.58
22.22
21.76
16.03
150 140 130 120 110 100 90 80 70 60 50 40 30 20 10 0 -10

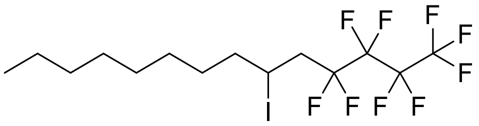
F F F
F
F
I F F F F

15r

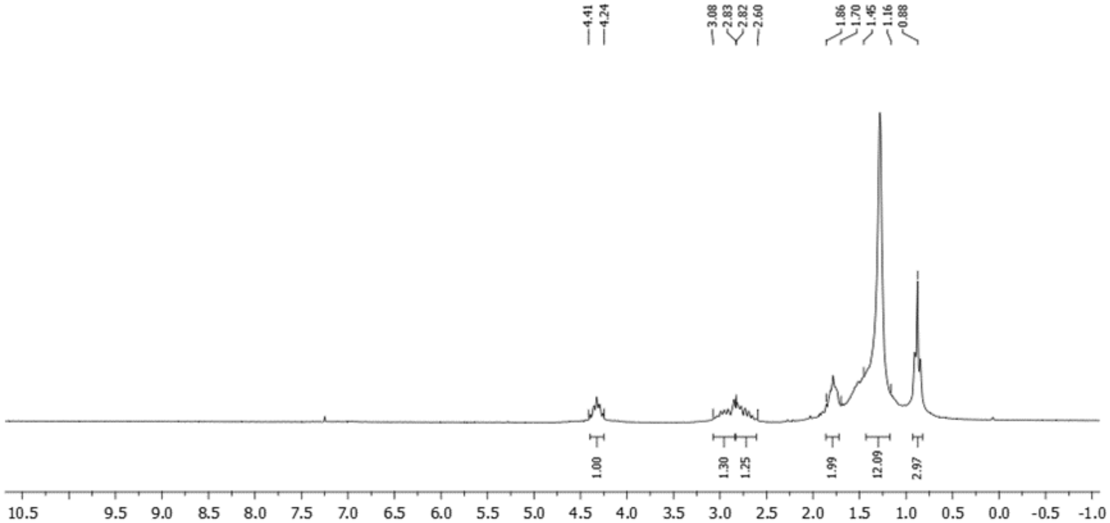
4.41
4.24
3.08
2.83
2.82
2.60
1.86
1.70
1.45
1.16
0.88
1.00
1.30
1.25
1.99
12.09
2.97
10.5 9.5 9.0 8.5 8.0 7.5 7.0 6.5 6.0 5.5 5.0 4.5 4.0 3.5 3.0 2.5 2.0 1.5 1.0 0.5 0.0 -0.5 -1.0

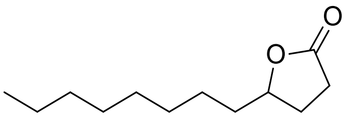
O
O

15s

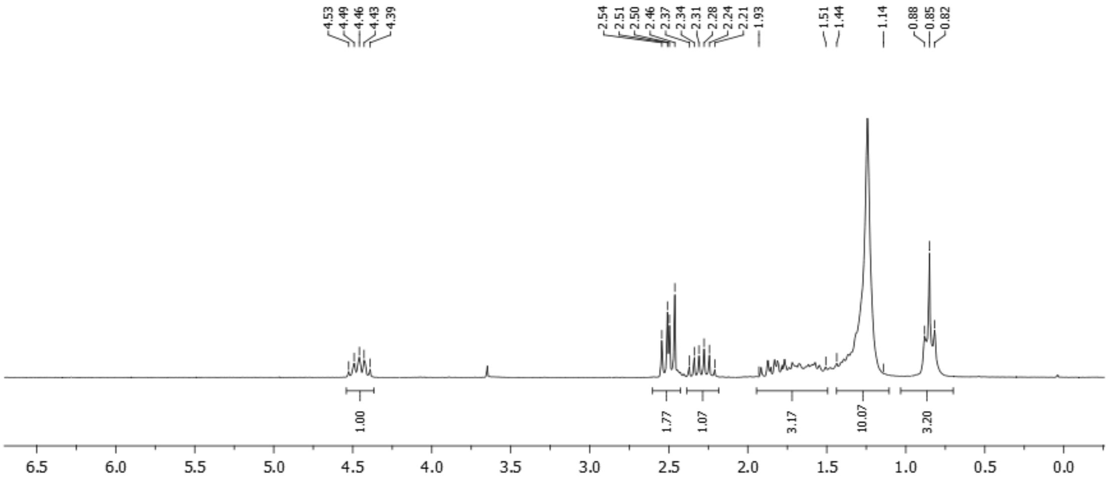
4.53
4.49
4.46
4.43
4.39
2.54
2.51
2.50
2.46
2.37
2.34
2.31
2.28
2.24
2.21
1.93
1.51
1.44
1.14
0.88
0.85
0.82
1.00
1.77
1.07
3.17
10.07
3.20
6.5 6.0 5.5 5.0 4.5 4.0 3.5 3.0 2.5 2.0 1.5 1.0 0.5 0.0

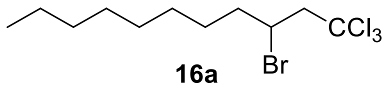
CCl3
Br

16a

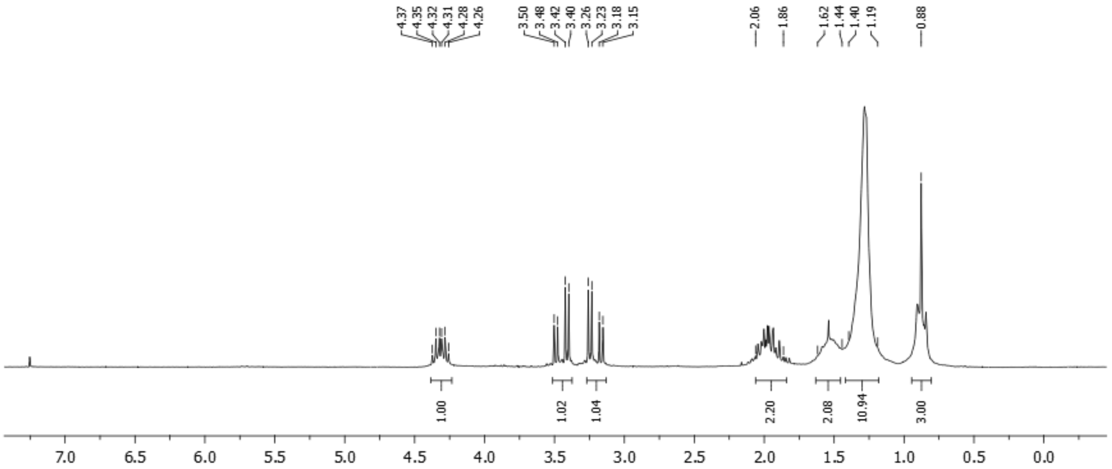
4.37 4.35 4.32 4.31 4.28 4.26
3.50 3.48 3.42 3.40 3.26 3.23 3.18 3.15
2.06 1.86 1.62 1.44 1.40 1.19 0.88
1.00 1.02 1.04 2.20 2.08 10.94 3.00
7.0 6.5 6.0 5.5 5.0 4.5 4.0 3.5 3.0 2.5 2.0 1.5 1.0 0.5 0.0

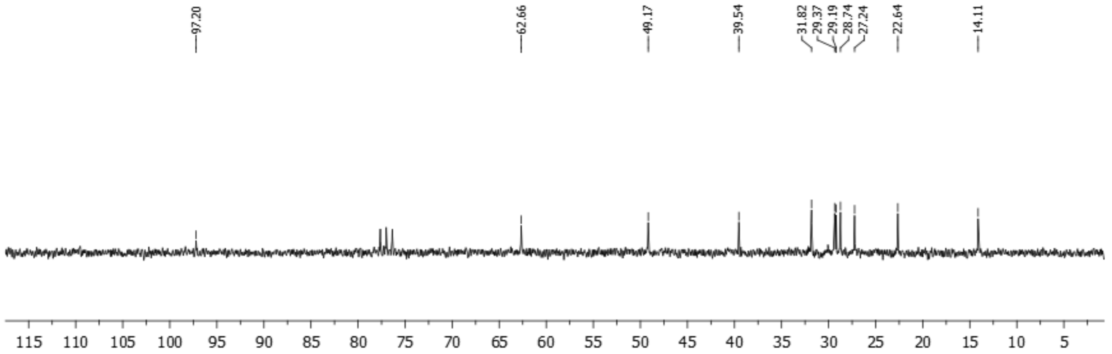
97.20 62.66 49.17 39.54 31.82 29.37 29.19 28.74 27.24 22.64 14.11
115 110 105 100 95 90 85 80 75 70 65 60 55 50 45 40 35 30 25 20 15 10 5

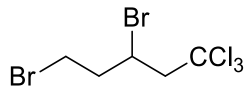
Br
Br
CCl3

16b

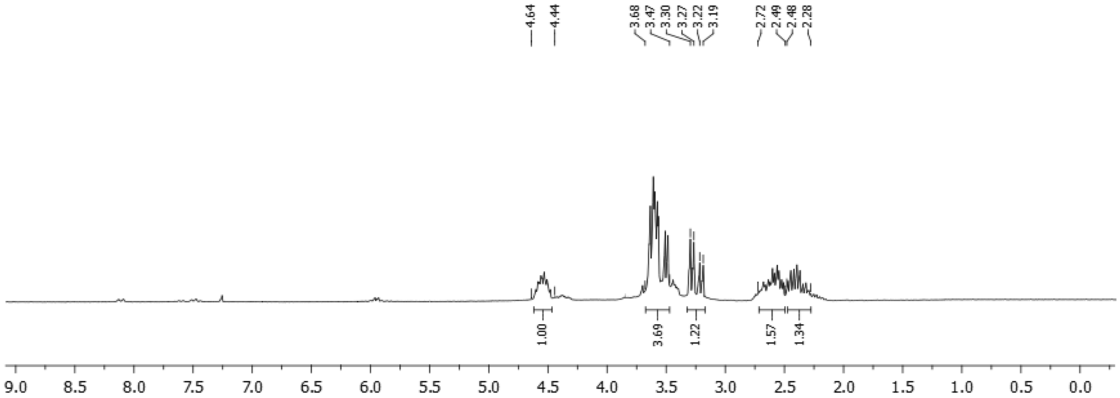
4.64
4.44
3.68
3.47
3.30
3.27
3.22
3.19
2.72
2.49
2.48
2.28
1.00
3.69
1.22
1.57
1.34
9.0
8.5
8.0
7.5
7.0
6.5
6.0
5.5
5.0
4.5
4.0
3.5
3.0
2.5
2.0
1.5
1.0
0.5
0.0

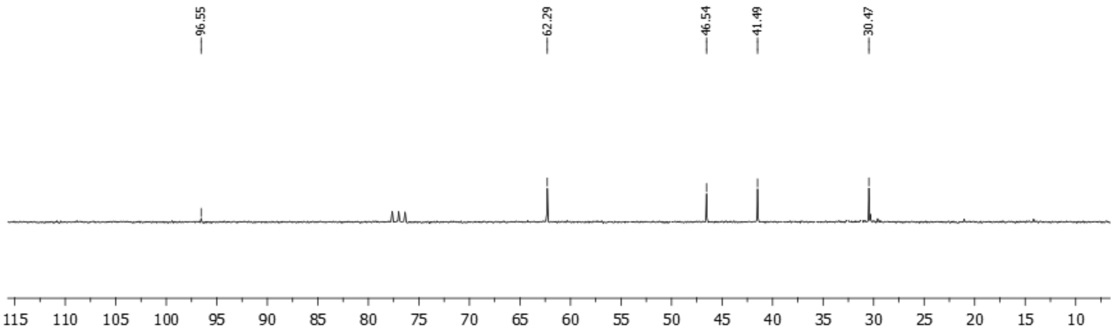
96.55
62.29
46.54
41.49
30.47
115
110
105
100
95
90
85
80
75
70
65
60
55
50
45
40
35
30
25
20
15
10

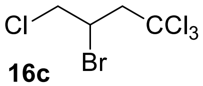
Cl
CCl3
Br

16c

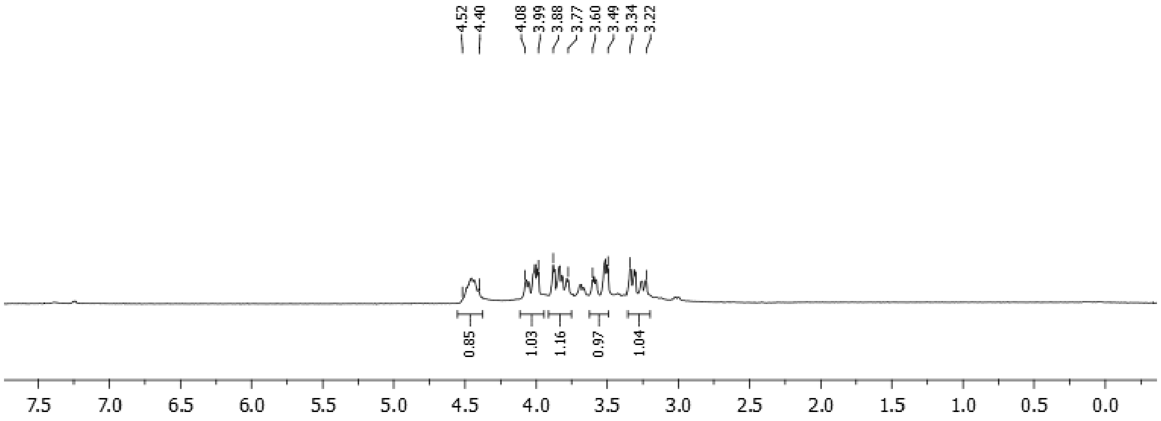
4.52
4.40
4.08
3.99
3.88
3.77
3.60
3.49
3.34
3.22
0.85
1.03
1.16
0.97
1.04
7.5 7.0 6.5 6.0 5.5 5.0 4.5 4.0 3.5 3.0 2.5 2.0 1.5 1.0 0.5 0.0

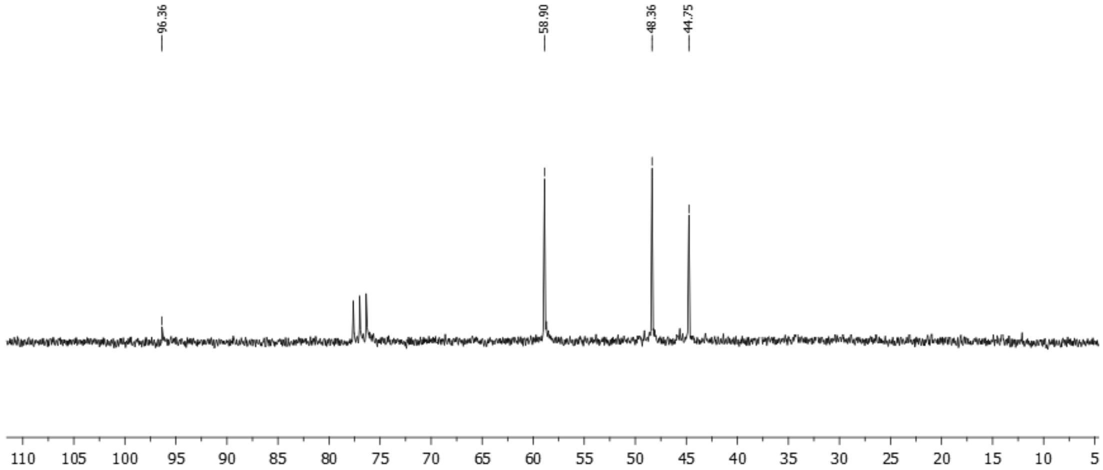
96.36
58.90
48.36
44.75
110 105 100 95 90 85 80 75 70 65 60 55 50 45 40 35 30 25 20 15 10 5

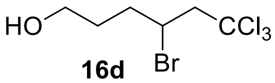
HO
CCl3
Br

16d

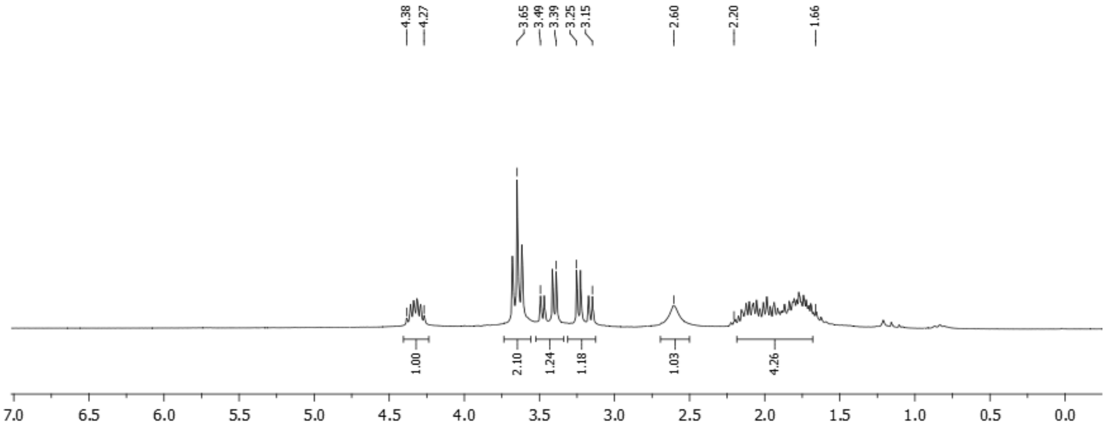
4.38
4.27
3.65
3.49
3.39
3.25
3.15
2.60
2.20
1.66
1.00
2.10
1.24
1.18
1.03
4.26
7.0
6.5
6.0
5.5
5.0
4.5
4.0
3.5
3.0
2.5
2.0
1.5
1.0
0.5
0.0

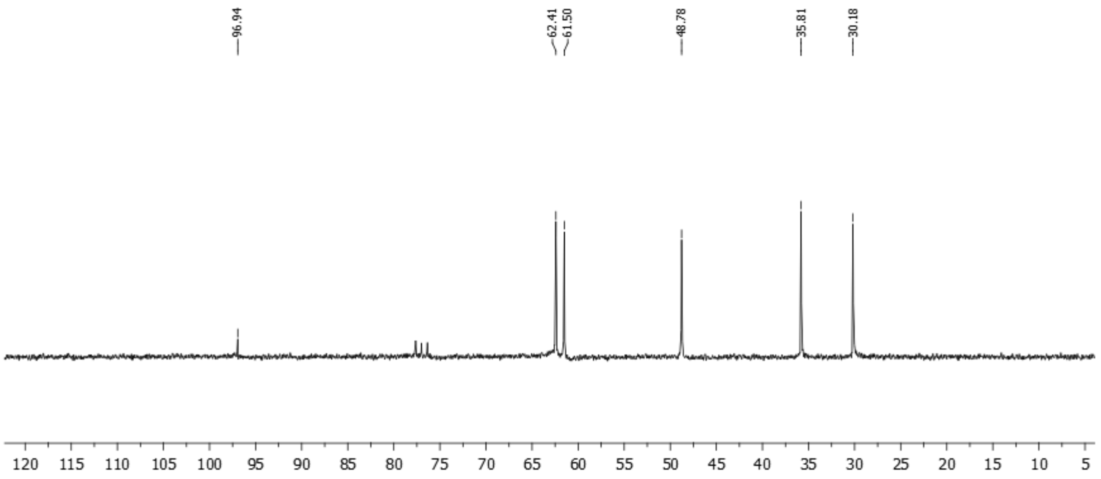
96.94
62.41
61.50
48.78
35.81
30.18
120
115
110
105
100
95
90
85
80
75
70
65
60
55
50
45
40
35
30
25
20
15
10
5

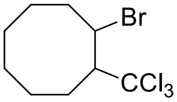
Br
CCl3

16e

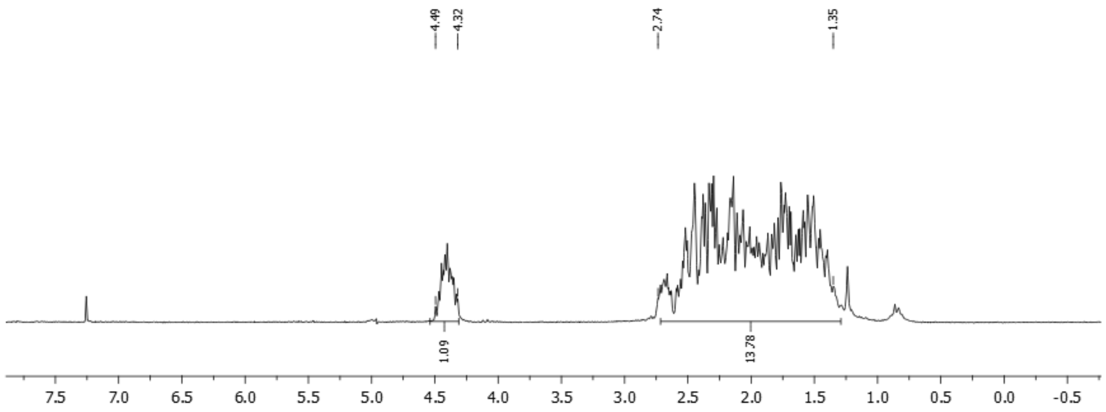
4.49
4.32
2.74
1.35
1.09
13.78
7.5 7.0 6.5 6.0 5.5 5.0 4.5 4.0 3.5 3.0 2.5 2.0 1.5 1.0 0.5 0.0 -0.5

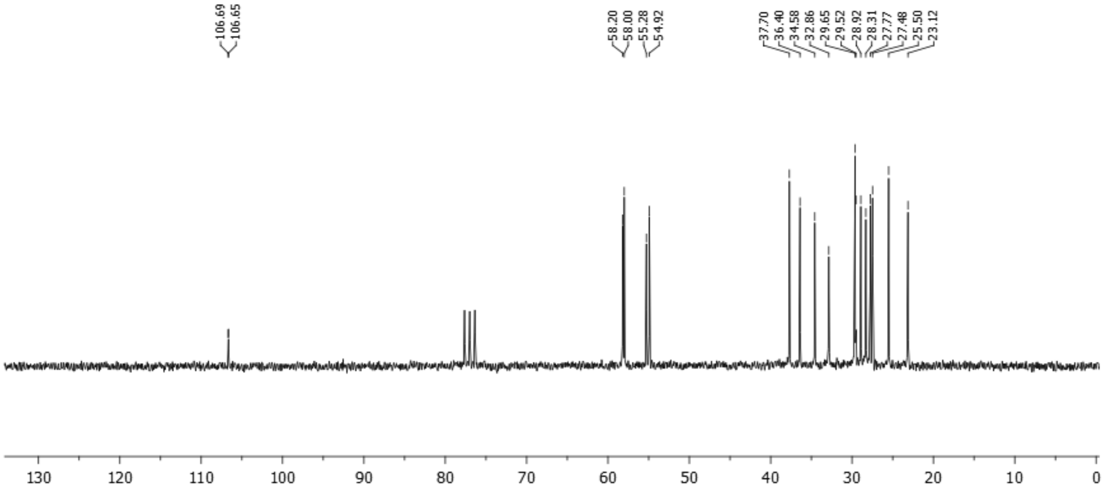
106.69
106.65
58.20
58.00
55.28
54.92
37.70
36.40
34.58
32.86
29.65
29.52
28.92
28.31
27.77
27.48
25.50
23.12
130 120 110 100 90 80 70 60 50 40 30 20 10 0

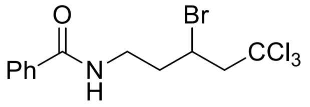
O
Br
Ph
N
H
CCl_3

16f

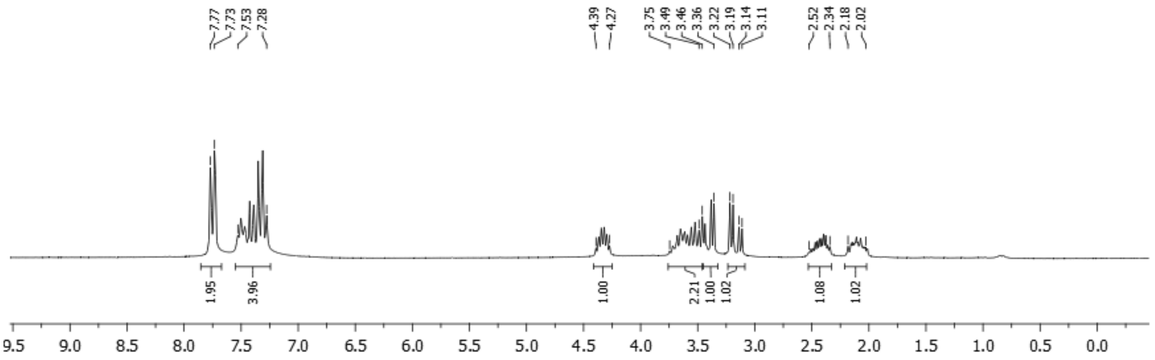
7.77
7.73
7.53
7.28
4.39
4.27
3.75
3.49
3.46
3.36
3.22
3.19
3.14
3.11
2.52
2.34
2.18
2.02
1.95
3.96
1.00
2.21
1.00
1.02
1.08
1.02

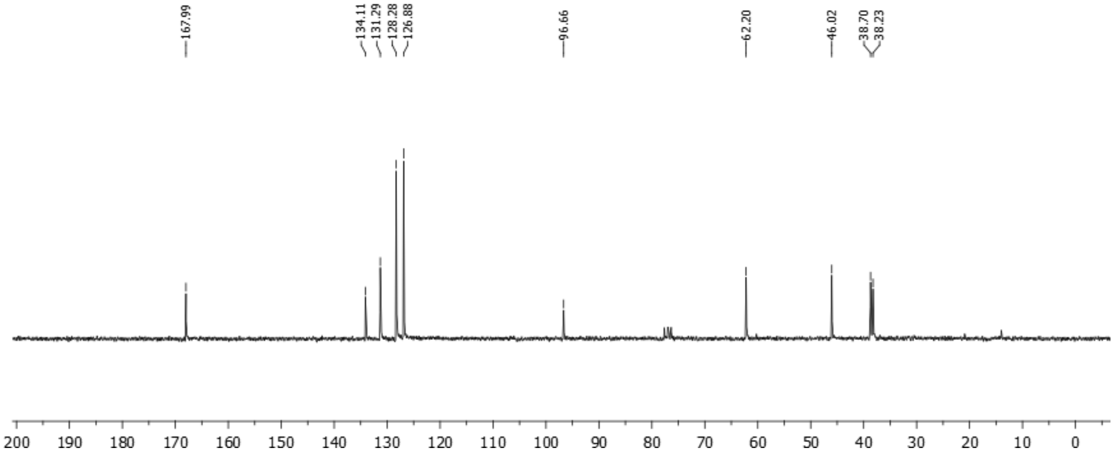
167.99
134.11
131.29
128.28
126.88
96.66
62.20
46.02
38.70
38.23

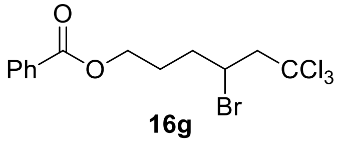
O
Ph
O
CCl3
Br

16g

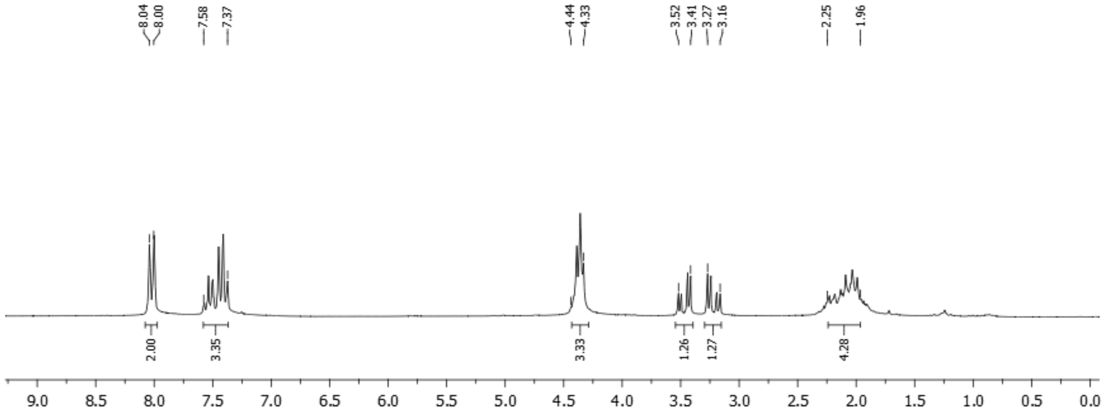
8.04
8.00
7.58
7.37
4.44
4.33
3.52
3.41
3.27
3.16
2.25
1.96
2.00
3.35
3.33
1.26
1.27
4.28
9.0 8.5 8.0 7.5 7.0 6.5 6.0 5.5 5.0 4.5 4.0 3.5 3.0 2.5 2.0 1.5 1.0 0.5 0.0

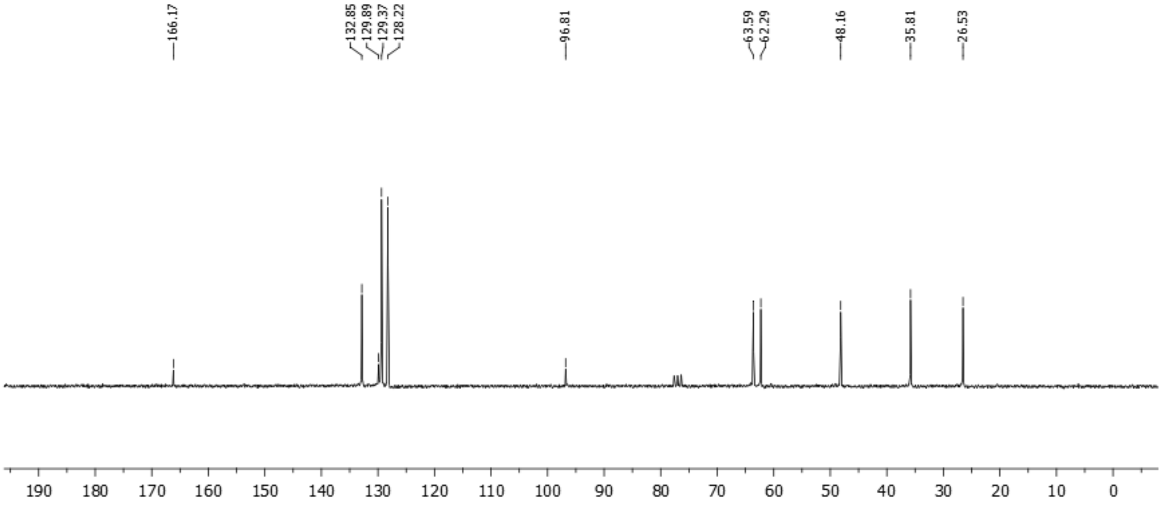
166.17
132.85
129.89
129.37
128.22
96.81
63.59
62.29
48.16
35.81
26.53
190 180 170 160 150 140 130 120 110 100 90 80 70 60 50 40 30 20 10 0
